# Magnetic Forces and DNA Mechanics in Multiplexed Magnetic Tweezers

**DOI:** 10.1371/journal.pone.0041432

**Published:** 2012-08-03

**Authors:** Iwijn De Vlaminck, Thomas Henighan, Marijn T. J. van Loenhout, Daniel R. Burnham, Cees Dekker

**Affiliations:** Kavli Institute of Nanoscience, Delft University of Technology, Delft, The Netherlands; Northeastern University, United States of America

## Abstract

Magnetic tweezers (MT) are a powerful tool for the study of DNA-enzyme interactions. Both the magnet-based manipulation and the camera-based detection used in MT are well suited for multiplexed measurements. Here, we systematically address challenges related to scaling of multiplexed magnetic tweezers (MMT) towards high levels of parallelization where large numbers of molecules (say 10^3^) are addressed in the same amount of time required by a single-molecule measurement. We apply offline analysis of recorded images and show that this approach provides a scalable solution for parallel tracking of the xyz-positions of many beads simultaneously. We employ a large field-of-view imaging system to address many DNA-bead tethers in parallel. We model the 3D magnetic field generated by the magnets and derive the magnetic force experienced by DNA-bead tethers across the large field of view from first principles. We furthermore experimentally demonstrate that a DNA-bead tether subject to a rotating magnetic field describes a bicircular, Limaçon rotation pattern and that an analysis of this pattern simultaneously yields information about the force angle and the position of attachment of the DNA on the bead. Finally, we apply MMT in the high-throughput investigation of the distribution of the induced magnetic moment, the position of attachment of DNA on the beads, and DNA flexibility. The methods described herein pave the way to kilo-molecule level magnetic tweezers experiments.

## Introduction

Magnetic tweezers (MT) are a powerful single-molecule technique for the study of the mechanics of macromolecules and the dynamics of enzymes that act on DNA or RNA [1], [2], [3]. In a typical MT assay, a DNA or RNA molecule is tethered between a paramagnetic bead and the surface of a flow cell. Tension and torque can be applied to the molecule by means of external magnets and the length of the tethered molecule is measured in real time by tracking the xyz-position of the paramagnetic bead using video microscopy, thereby providing a means for monitoring, for example, enzyme-induced changes in the length and topology of the molecule.

MT have provided unique insights into the activity of polymerases [4], helicases [5], recombinases [6], [7] and topoisomerases [8] on DNA. Key advantages of MT include the simplicity and low cost of its implementation, and the ability to study the influence of tension [9] and torque [10] on enzymatic activity. Both the magnetic-force-based manipulation and the camera-based detection used in MT are compatible with multiplexing [11], [12], [13], [14]. Multiplexing has provided greatly increased data-throughputs in single molecule experimentation based on e.g. centrifugal forces [15] and DNA-force probes [16]. The implementation of multiplexing in MT is made difficult by a number of challenges related to e.g. non-uniformities in the applied force field and the challenge of implementing highly parallel position tracking and DNA-molecule characterization. Here, we systematically investigate these aspects and we present solutions to various issues encountered.

We first address the computational challenge related to tracking the xyz-positions of many beads (i.e. >100). We apply an offline analysis of recorded images and show that this approach provides a scalable solution. We use a large field-of-view optical imaging system (300×400 µm) to maximize the number of DNA-bead tethers addressed in experiments. We model the 3D magnetic field generated by the external magnets and compute the magnetic force exerted on DNA-bead tethers across the large field of view. We show that the magnetic force is not uniformly distributed and varies with the position of the DNA-bead tethers when the magnet is in close proximity of the flow cell (up to 24% variation in magnitude of force across the field). We furthermore demonstrate that the angle of the force vector can be extracted experimentally from magnet-rotation measurements. We show that a DNA-bead tether subject to a rotating magnetic field, describes a bicircular rotation pattern that is well described in terms of a Limaçon roulette. Both the angle of the force exerted on the DNA-bead tether and the position of attachment of the DNA on the bead can be accurately extracted by analyzing this Limaçon rotation pattern. A thorough understanding of the 3D distribution of the force fields and the possibility to directly extract information about the angle and magnitude of the force vector in experiments will allow researchers to design MMT with larger fields of view, leading to a higher experimental throughput.

Next, we address DNA-molecule characterization and selection in the context of MMT. In a standard single-molecule MT assay, molecules are selected after characterization of the mechanical properties. In particular, DNA-bead tethers are selected that display a full length that corresponds to the expected length of the molecule, whereas tethers that are shorter due to an eccentricity of the point of attachment of the DNA to the bead are avoided. In MMT, it is not desirable to carry out such molecule selection, and as many single molecules tethers as possible should be included in the analysis. We describe techniques for the high-throughput characterization of the mechanical properties of the molecules, including the DNA attachment point on the bead, and the DNA persistence and contour lengths. We apply MMT to the large-throughput analysis of DNA mechanics and we investigate the dependence of the measured mechanical properties of dsDNA molecules on the molecule length. We furthermore provide high-throughput measurements of the distribution of the magnetic moments induced in the paramagnetic beads and we analyze the distribution of DNA-bead-attachment positions.

All in all, the methods and guidelines presented here will enable the design of high-throughput MMT experiments. Together with the development of a method for the directed tethering of DNA-bead tethers in regular arrays leading to a higher density of DNA-bead tethers in the field of view [17], the results and methods presented here pave the way for routine kilo-molecule MT experiments.

## Materials and Methods

### DNA Substrates

DNA substrates with different lengths (2.2 kb, 7.3 kb, 11.9 kb and 20.1 kb) were prepared carrying multiple digoxigenin labels at one end and a single biotin label on the other end, thereby following a previously described procedure [6]. The resulting DNA-bead tethers were torsionally unconstrained.

### Magnetic Tweezers

A magnetic tweezers setup was used in these experiments as described [17]. A 1.4 megapixel camera (Dalsa, Falcon 1.4M100) was used in this work. The optical imaging system has a 25× magnification and consists of a 50×oil immersion objective (CFI Plan 50XH Nikon) and a 100 mm tube lens (PAC073, Newport). The resulting field of view size is 300 µm×400 µm. Vertical translation and rotation of the magnet pair are respectively achieved with a translation DC motor (M-126.PD2, Physik Instrumente) and rotation DC motor (C-150, Physik Instrumente). Bead-DNA tethers are created in a flow cell using a previously described protocol [18]. The flow cell consists of two cover glasses (thickness 0.17 mm) joined by a double layer of molten parafilm (thickness 0.23 mm).

In experiments, the xyz-positions of 5 magnetic beads are measured in real-time for direct monitoring of the response of a subset of the bead-DNA tethers in the field of view and images with information on all beads are saved to a hard drive (Western Digital, WD1001FALS) after JPEG compression (JPEG quality factor 900, image save time 12.2 ms, unless stated otherwise). The saved images are analyzed after the experiment to extract the time-dependent position of all beads in the field of view. To exclude effects of thermal drift, positions were measured relative to a non-magnetic polystyrene bead (Bang Laboratories, Carmel, IN) fixed to the bottom of the flow cell. Programs for online and offline image analysis were written in LabVIEW. A stand-alone version of the program used for image post analysis can be downloaded from the website of the authors: http://ceesdekkerlab.tudelft.nl/download. All measurements were carried out at 22°C and in 20 mM Tris-HCl, pH 7.5 buffer. We used streptavidin-coated superparamagnetic beads with a diameter of 1.05 µm (Dynal MyOne beads, Invitrogen, Carlsbad, CA). The number of trackable DNA-bead tethers in the field of view depends on the minimum bead-to-bead separation that can be tolerated and the field of view size. In case of random tethering and a minimum bead-to-bead separation of 15 µm, a 300×400 µm sized field of view produces a maximum of 60 productive DNA-bead tethers (see eq. 1 in ref. [17]).

### 3D Force Field Modeling

Forces are exerted using an external magnet pair. The magnetic moments of the magnets were oriented antiparallel with respect to each other and perpendicular to the flow cell surface. The magnet-to-magnet distance was 2 mm. We have numerically calculated (Mathematica 7.0) the 3D magnetic field distribution and the magnitude and orientation of the forces exerted on superparamagnetic beads as function of distance between the bottom surface of the magnet pair to the top of the flow cell, Z_mag_. A detailed description of the formalism used can be found in the supplementary methods (Methods S1). The source code and the force field calculations listed in this work can be found on the website of the authors: http://ceesdekkerlab.tudelft.nl/download.

### Modeling of Dynamic Force Response

We have investigated the response of a DNA-bead tether to a time-dependent applied magnetic force using numerical simulations. The model is based on a finite-difference approximation to the dynamic equation of motion of a DNA-bead tether and was implemented in MATLAB (The MathWorks, Natick, MA). A detailed description of the model can be found in the Supplementary methods (Methods S1) along with comparisons of the model predictions with experimental data (see Fig. S2).

## Results and Discussion

### Off-line Image Analysis for Scalable MMT

**Figure 1:**
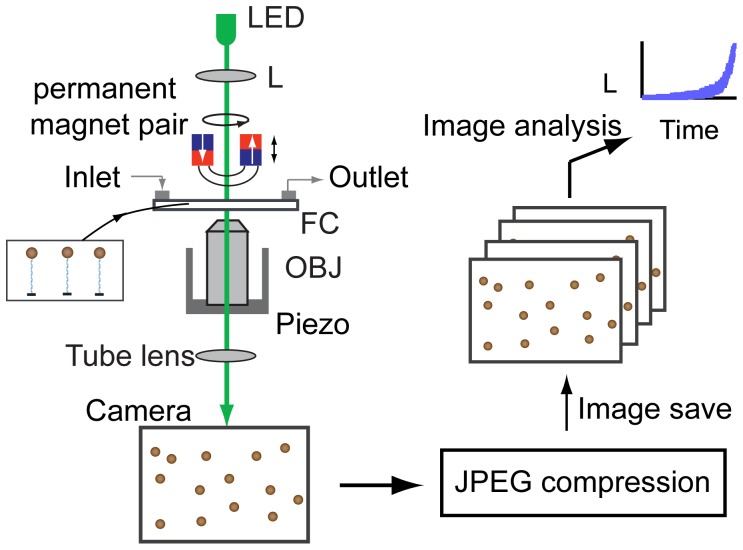
Principle of magnetic tweezers (MT) and increase in data throughput via parallelization. Positioning and rotation of an external magnet pair allows application of a force and torque to DNA-molecules bound to paramagnetic beads. DNA-bead tethers in the flow cell (FC) are visualized using a microscope system, consisting of an LED, a lens (L), an objective (OBJ), a tube lens and a camera. Images of the experiment are JPEG-compressed and saved to a hard drive. After the experiment, the images are analyzed and the xyz-positions of all beads in the field of view are extracted.

We first address the difficulty of tracking of the xyz-positions of many DNA-bead tethers without compromising the position resolution or acquisition frame rate. Real-time tracking has been demonstrated for up to 34 beads in parallel at a 60 Hz frame rate [11] but becomes cumbersome when a large number of beads (>100) need to be tracked or when a higher frame rate is desirable. Here we bypass the computational challenge of real-time position tracking by storing images to a hard drive and analyzing the images after the experiment (see Fig. 1), as in [15]. All images were analyzed using the Quadrant-Interpolation (QI) position-tracking algorithm described in [19]. The QI algorithm provides a higher tracking resolution than standard algorithms used for position tracking in MT, particularly at low magnification, but is computationally more demanding. Images are stored after image compression to reduce the image size and save time. We compared TIFF, PNG and JPEG based compression algorithms and found that JPEG compression provides the best compromise between image save time and tracking resolution (data not shown). We have investigated the trade-off between the compression-level-dependent save-time and resolution in tracking and find that JPEG compression allows saving images at a high rate (>50 Hz) with minimal loss of resolution in tracking (see Fig. S1).

### Quantitative 3D Modeling of the Magnetic Field and Force Distribution

The number of molecules that can be investigated in parallel in MMT can readily be increased by enlarging the effective field of view size of the optical imaging system, e.g. by employing a camera with a larger imaging array. The assumption routinely made in magnetic tweezers experiments that the force is constant over the field of view breaks down if the field of view is similar or larger in size than the characteristic length over which the magnetic field varies. Here, we model the magnetic field and force distribution across the field of view for the magnet configuration used in this work. Furthermore, we introduce a method that allows extraction of the orientation of the force vector in experiments.

We have numerically calculated the 3D magnetic field distribution and the corresponding magnitude and orientation of the force exerted on a superparamagnetic bead as function of the distance between the DNA-bead tether and the magnet pair. A detailed description of the formalism used can be found in the Methods S1. Briefly, the magnetic force exerted on the paramagnetic bead is calculated using 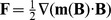, as in Ref. [20], where 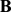 is the external magnetic field and 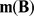 is the magnetic moment induced in the paramagnetic bead. In analogy of field calculations in electrostatics, the magnets are modeled as two fictitious magnetic charge sheets located at the bottom and top planes of the magnets (with uniform magnetic charge density ρ_M_, see Fig. S6), and the magnetostatic fields generated by the magnets are calculated using a magnetostatic analog of Poisson’s equation [21]. The induced magnetic moment in the paramagnetic beads is described by a Langevin function, 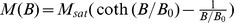, where M_sat_ and B_0_ are the saturation magnetization and the characteristic field of the superparamagnetic beads respectively (M_sat_ = 43.3 kA/m and B_0_ = 12 mT quoted by the vendor).

We first used the above formalism to calculate the magnetic field and vertical force experienced by a magnetic bead aligned to the rotation axis of the magnet pair. Fig. S6 shows the predicted magnetic field strength as function of the magnet height. We compared the calculated field strength to results from Hall probe measurements described in [20], and found a good agreement between model results and measurement data (Fig. S6b). Next, we calculated the resulting vertical magnetic force, F_mag,z_, experienced by a paramagnetic bead aligned to the rotation axis of the magnet pair. We compared the data with results from 2D calculations and finite-element-based modeling [20] and again find a good agreement (see Fig. S6). Next, we compared calculations of F_mag,z_ with data obtained in MMT measurements and find a good agreement (Fig. S8). The measured and simulated force are exponentially dependent on the magnet height for magnet positions Z_mag_>1 mm with a force decay length of 1.86 mm predicted by simulations and 1.54 mm observed in experiments. The discrepancy between measured and simulated forces is likely explained by the finite magnetic polarizability of the objective and the flow-cell mount that was not taken into account in the model.

**Figure 2:**
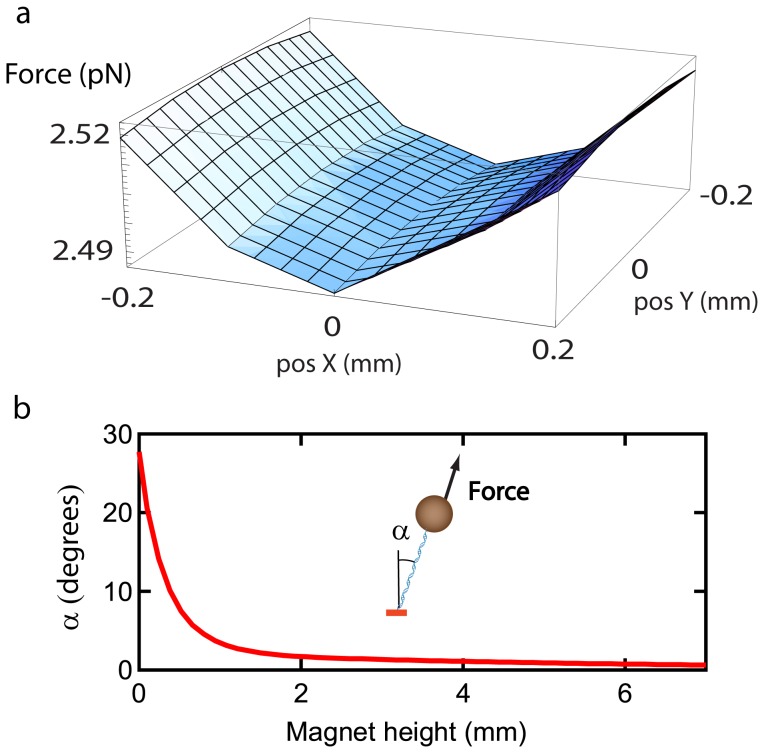
3D modeling of the variation of the force across the field of view and angle of the force vector. **A** Magnitude of force, F_mag_, acting on paramagnetic beads as function of their position in the field of view (field of view size = 400×400 µm) for a magnet height of 1 mm (symmetry axis of the magnet pair parallel to y-axis). A maximum variation in the force magnitude of 1.2% is found. **B** Angle between the force vector and the vertical axis, α, for a bead-DNA tether at the edge of the field of view (position in x, posX = 0.2 mm, position in y, posY = 0 mm). α = 27.7° for Z_mag_ = 0 mm and α = 3.4° for Z_mag_ = 1 mm.

Having established that the model described herein faithfully predicts the magnetic force and field, we calculated the distribution of the vertical force, F_mag,z_ as function of the position of the bead in the field of view. Figure 2 shows the distribution for a distance of the bottom of the magnet pair to the top surface of the flow cell of Z_mag_ = 1 mm. The graph shows a maximum variation in the magnitude of the force of 1.2% for a field of view of 400×400 µm. For Z_mag_ = 0 mm and Z_mag_ = 2 mm, we find a variation of 24% and 0.3% respectively (see Fig. S6). The F_mag,z_ variation across the field of view is accompanied with a finite lateral force, F_mag,x_. Figure 2b shows the angle of the force vector to the vertical axis, 
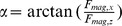
 as a function of Z_mag_ for the case of a DNA tethered bead at a distance of 200 µm to the rotation axis (α = 0.36 rad ≈ 28° for Z_mag_ = 0 mm).

This analysis shows that for Z_mag_<1 mm and the magnet configuration used in this work, the force is not constant across the field of view (i.e. varies with more than 1.2%) and an appreciable lateral force is exerted on DNA-bead tethers at the extremity of the field of view. In the measurements of the force response of DNA-bead tethers described below, we have used Z_mag_>1 mm. A thorough understanding of the force fields will be useful in the design of future MMT assays with larger fields of view that will benefit from a higher experimental data-throughput, for example by using a larger imaging camera. Below, we describe a method for the extraction of the angle of the force vector through a magnet-rotation measurement, thereby facilitating a complete description of the field variations in experiments.

### Bead DNA Tether Describes a Limaçon Rotational Pattern

**Figure 3:**
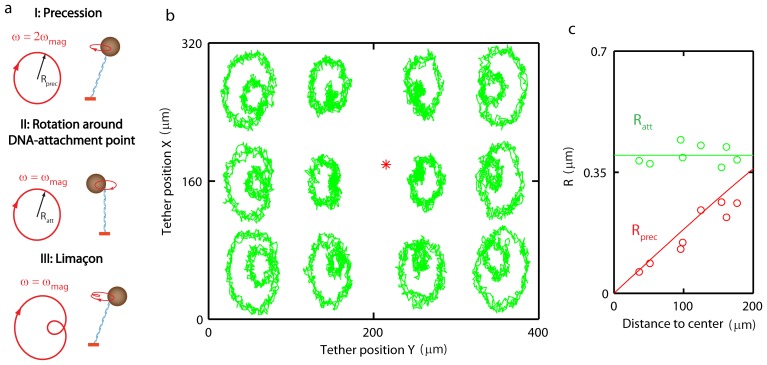
Rotational response of a DNA-bead tether. **A** The response of a DNA-bead tether subject to a rotating external magnet pair (frequency of rotation ω_mag_) that is misaligned with respect to the axis of rotation of the magnet pair and for which the DNA attachment position is eccentric, is a combination of a precessional motion with radius, R_prec_ and frequency ω = 2ω_mag_ (panel I) and a rotational motion around the DNA attachement point with radius, R_att_ and frequency ω  =  ω_mag_ (panel II). The bi-circular rotation pattern describes a roulette that is a special case of the epitrochoid: the Limaçon (panel III). **B** Measurement of the rotational response for a DNA-bead tether at different positions in the field of view The * denotes the position of the rotation axis of the magnet. For clarity in presentation, the patterns were scaled up by a factor 37.5 compared to the scale of the field of view. **C** R_att_ and R_prec_ as function of the distance of the tether position to the center of the magnet. R_att_ is independent of the position of the tether in the field of view, as expected. R_prec_ increases with the distance to the center of the magnet. Red line is prediction based on calculations of the 3D force field (as in Fig. 2).

As a consequence of the non-uniform force distribution, a DNA-bead tether that is not exactly located midway between the magnet pair experiences a finite lateral force. A DNA-bead tether that is not aligned to the rotation axis of the magnet pair undergoes a precessional motion when subject to a rotation of the external magnets. Interestingly, half a turn of the magnet pair gives rise to a magnetic field with opposite polarity but the same field intensity distribution, and the resulting magnetic force acting on the bead is the same. It can thus be seen that the DNA-bead tether undergoes a full-circle precession in response to half a turn of the magnet. In general, if the magnet pair is rotated with frequency ω_mag_, the bead undergoes a precessional rotation with frequency ω_prec_ = 2ω_mag_ (see Fig 3a, panel I, and video S1). For small α, the precessional motion leads to a circular motion in the xy- plane.

A second, independent contribution to the overall rotational response of a DNA-bead tether is due to the random location of the point of attachment of the DNA to the bead [22]. The position of attachment of the DNA on the surface of the bead is relevant given that the magnetic bead has a non-uniform paramagnetic polarizability [22]. In the presence of a magnetic field, the paramagnetic bead experiences a torque that attempts to align the easy axis of the paramagnetic polarizability along the direction of the magnetic field (which is approximately parallel to the surface of the flow cell). We assume that for the forces applied in our experiments (F_mag,z_<2 pN), the torque due to alignment of the magnetic easy axis of the bead is much larger than the torque exerted by the vertical force, F_mag,z_, on a lever with length, l ≤ R_bead_, where R_bead_ is the radius of the bead [22]. The restoring torque thus prevents the out-of-plane rotation of the bead and the location of the DNA-attachment on the bead in the presence of a magnetic field can therefore differ from the geometric bottom of the bead. An eccentric DNA-attachment location (see above) gives rise to a circular rotation around the attachment position upon rotating the external magnets (see Fig. 3a, panel II, and video S2). The rotational response is circular in the plane parallel to the surface and has a rotational frequency ω_att_ = ω_mag_.

The rotational response of a DNA-bead tether that is misaligned with respect to the axis of rotation of the magnet pair and for which the DNA attachment position is eccentric, is thus a combination of a precessional motion with ω_prec_ and a rotational motion ω_att_, where ω_prec_ = 2ω_att_. The combined, bi-circular motion gives rise to a rotational curve in the plane parallel to the surface that is an example of an epitrochoid curve, the curve that is similarly traced by a point attached to a circle that rolls about the outside of a second circle [23]. It can be seen that frequencies of rotation and rolling of the rolling circle differ by a factor of two when the static and rolling circle have an equal radius. Roulettes for which this condition is met are a special case of the general class of epitrochoid curves called Limaçon. In Cartesian coordinates, the centered rotation pattern is described by:


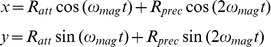
(1)

where R_att_ is the radius of the circle described due to a rotation around the DNA-bead attachment point and R_prec_ is the radius of rotation due to the precessional rotation. For 2 R_prec_> R_att_>0, the curve crosses itself forming an inner loop (Fig. 3a, panel III, and video S3).

We have analyzed the rotational pattern of a DNA-bead tether (20 kb dsDNA) at different locations in the field of view. Indeed, the rotational patterns recorded at 12 different locations shown in Fig. 3b display a Limaçon pattern, indicative of a bi-circular response. The data were fit using eq. 1 to extract both the radius of precession, R_prec_, and the radius of rotation around the position of attachment of the DNA on the bead, R_att_. Fig. S10 shows the time-dependent response of a DNA-bead tether for the examples of a mono-circular response (R_prec_ ≈ 0) and a bi-circular response, for which a single and double frequency contribution is observed respectively. Fig. 3c shows R_att_ and R_prec_ as function of the distance to the magnet’s rotational axis, d_center_, recorded for Z_mag_ = 1 mm. The center of the magnet was extracted by analyzing the position dependence of R_prec_ and is indicated in Fig. 3c with an *. It is clear from Fig. 3c that R_att_ is independent of d_center_, as would be expected. The dependence of R_prec_ on d_center_ was compared to results from numerical simulations (using the formalism described above), and a good agreement between experiments and simulations. Fig. S11 shows the measured and expected dependencies of R_prec_ on d_center_ for different magnet heights (Z_mag_ = 0.2 mm, Z_mag_ = 0.5 mm and Z_mag_ = 1 mm). The R_prec_ depends stronger on d_center_ for a lower magnet height as would be expected from the 3D force-field calculations that show a stronger variation in the force field for low Z_mag_.

### DNA Characterization: Bead Attachment Offset and Molecule Length

In single-molecule MT, the mechanical properties of a selected molecule are characterized prior to subsequent DNA-enzyme interaction experiments. Typically, a molecule is selected that displays a maximum end-to-end distance close to the expected contour length of the molecule, whereas molecules with large bead attachment offset and multiply tethered beads are discarded. In MMT, ideally all singly tethered molecules are used in the further analysis to maximize the experimental throughput. There is thus a need for an accurate and high-throughput characterization of the mechanical properties of the molecules, including the length, the bead-attachment position and the persistence length of the molecule. Here we describe an automated analysis protocol that allows faithful extraction of these parameters in under ten minutes of measurement time.

**Figure 4:**
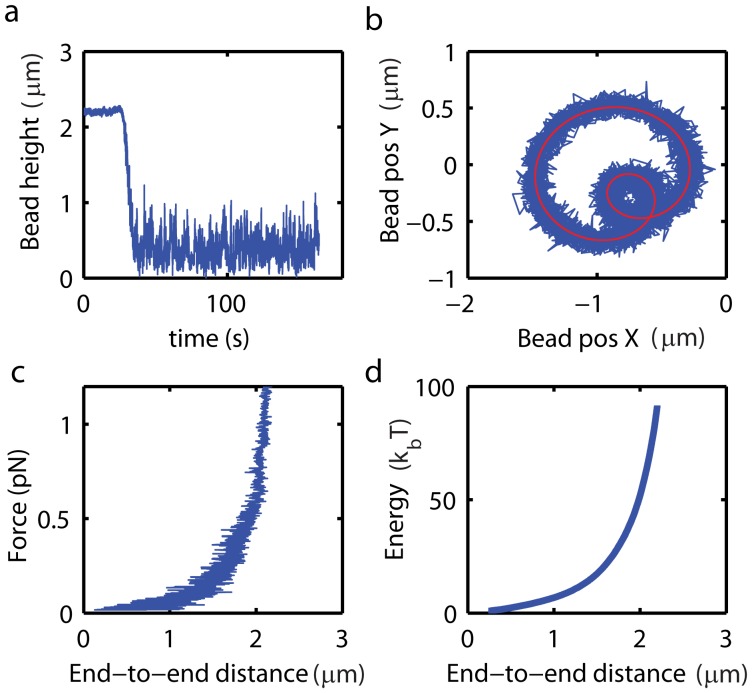
Fact characterization of the mechanical properties of a DNA-bead tether. A Characterization of the DNA length. The lowest bead height in the absence of an applied force is measured and the length is accordingly offset corrected. **B** Measurement of bead attachment offset from rotational response of the DNA-bead tether (cf. Fig. 3). **C** Dynamic measurement of the force-extension characteristic of the molecule. The length of the molecule was measured while linearly increasing the magnet height. The applied vertical magnetic force, F_mag,z_, is exponentially dependent on the magnet height (see sup Fig. S7). 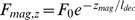. F_0_ is a force-calibration factor that was characterized in an independent measurement to account for bead-to-bead variations in magnetic polarizability. **D** Energy versus extension curve allows extracting values for the persistence length and contour length with high fidelity [4]. Energy versus extension was calculated from the data in panel c using eq. (2).

In the first step of this protocol, the length of the molecules is characterized. To this end, the length of the molecule at the maximum force used in the analysis (∼2 pN, corresponding to Z_mag_ = 1 mm) is measured for a duration of 25 s. Subsequently, the magnet is moved upward to Z_mag_ = 20 mm, for which the applied force becomes vanishingly small (F_mag,z_<0.1 fN). The fluctuations of the bead in the absence of applied force are recorded over 130 s. The lowest recorded height in the absence of force then corresponds to the apparent height of the bead when in contact with the surface of the flow cell, and the data is accordingly height-offset corrected. See Fig. 4a for an offset-corrected length measurement trace recorded for a 7.3 kb dsDNA.

**Figure 5:**
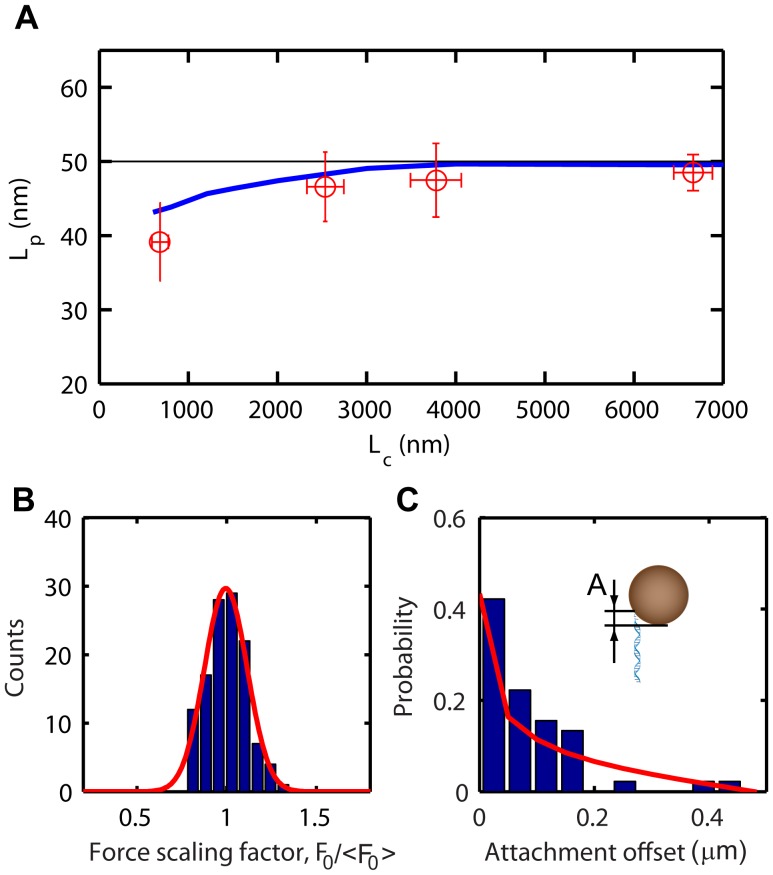
Ensemble analysis of DNA mechanics. **A** Multiplexed measurements of the persistence length, L_p_, versus the contour length, L_c_, for DNA molecules with different length (2.2 kb (N = 19), 7.3 kb (N = 16), 11.9 kb (N = 36) and 20.1 kb (N = 9)). The data is compared to predictions from finite-difference modeling in the absence of thermal fluctuations (black line) and in the presence of thermal fluctuations (blue line). **B** Histogram of extracted force-calibration factor F_0_/<F_0_> (N = 120). **C** Ensemble study of attachment position of the DNA on the bead. The histogram shows the distribution of DNA-bead attachment offsets (N = 45). Red line is the expected distribution (see Fig. S5 and eq. 3).

In the next step, we characterize the length offset due to the position of attachment of the DNA on the bead. An eccentric location of the DNA-attachment point, deviating from the bottom of the bead, gives rise to a discrepancy between the measured bead height and the actual end-to-end distance of the molecule [22]. Using numerical simulations of the force response of a bead-DNA tether, we have investigated the effect of this discrepancy on force-extension experiments, and we find that an eccentric DNA attachment point on the bead can lead to an underestimation of the measured persistence length by up to 13 nm (i.e. 25%) for a molecule with contour length L_c_ = 800 nm (see Fig. S4). It is therefore important that the length offset due to the bead-attachment position is well characterized, which we achieve here by analyzing the rotational response of the bead-DNA tether as described above. Fig. 4b shows the rotational pattern recorded upon rotating the external magnet for an individual DNA-bead tether (7.3 kb dsDNA). The length offset due to an eccentric position of attachment of the DNA on the bead, A (see Fig. 5c), is calculated from the measured bead-attachment rotation radius, R_att,_ 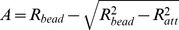, and is used to correct the measured force-response of the DNA molecule.

### DNA Force-response

The dependence of the DNA end-to-end length on force yields information about the mechanical properties of the molecule and allows distinguishing singly and multiply tethered beads. The force required to extend a dsDNA molecule, thereby reducing the conformational entropy of the molecule, is well described by the worm-like chain (WLC) model [24], [25]. The model has two parameters, the contour length of the molecule, L_c_, and the persistence length, L_p_, a measure for the length scale over which bending fluctuations decay. dsDNA has a well-defined L_c_ (0.34 nm per base pair) and L_p_ (L_p_ ≈ 50 nm where L_p_ is modestly dependent on salt concentration) [25].

In order to extract the force response of the DNA tethers, we measured the end-to-end distance of the molecule as function of the position of the magnet. Here, the magnet was moved upward at a constant rate (dZ_mag_/dt = 0.1 mm/s) giving rise to an exponentially decreasing applied force (see below). We have taken care that the corresponding rate of decrease in bead height is sufficiently slow such that the drag force, F_drag_, experienced by the bead can be disregarded (F_drag_<1 fN, F_drag_/F_mag,z_ <0.04, see Fig. S3). Force-field calculations indicate that the applied force is to good approximation exponentially dependent on Z_mag_, for Z_mag_ in the range 1 mm–8 mm (relative error <10% and absolute error <20 fN, see Fig. S7). Single-exponential behavior is assumed with 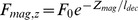, where F_0_ is a bead-specific force-calibration factor that is experimentally determined to account for bead-to-bead variations in induced magnetic moment and l_dec_ is the force decay-length. The force experienced by the bead was measured at three magnet positions by analyzing the Brownian noise spectrum of the bead [26]. The extracted force-calibration factor then allows to quantitatively predict the forces for all magnet positions, 1 mm Z_mag_<9 mm [20]. We experimentally determined a force decay length, l_dec_, = 1.54 mm, for the magnet configuration used in this experiment (Fig. S8). The data in Figure 4c shows a force versus end-to-end-distance curve (a 7.3 kb dsDNA) obtained after correcting for the bead-specific force calibration factor, F_0_ = 6.0 pN, and the DNA-attachment length-offset, A = 0.01 µm.

Subsequently, we follow the procedure proposed by Kruithof *et al.* to analyze the force response and extract the *L_p_* and *L_c_* of the molecule [27]. Here, the energy required to stretch the molecule to a given end-to-end distance, *l_ext_*, is calculated on the basis of the measured force-extension data:


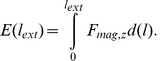
(2)

Fig. 4d shows the energy versus end-to-end distance obtained for the force response data plotted in Fig. 4c. The energy versus extension curve is much smoother than the original force-extension data as fluctuations in bead position are averaged out by the integration. We found that reordering the data prior to integration in such way that the extension increases monotonically, removes scatter from the data, ultimately allowing for more accurate determination of the mechanical parameters (Fig. S9) (parameters extracted in this example: L_p_ = 48.5 nm, L_c_ = 2.5 µm).

### Ensemble Analysis of DNA Mechanics

Using the procedure outlined above, we have analyzed the mechanical parameters of DNA-bead tethers. Figure 5a shows the persistence lengths plotted versus contour length extracted from multiplexed MT experiments on dsDNA molecules with 4 different lengths (2.2 kb (N = 19), 7.3 kb (N = 16), 11.9 kb (N = 36) and 20.1 kb (N = 9)). In the classic WLC model, L_p_ is a length-independent material property. The experimental data in Fig. 5a however shows a length-dependent L_p_, with L_p_ decreasing for shorter molecules. Fig. 5a shows the mean of the measured distributions; the error bars correspond to the standard deviation of measured persistence and contour lengths. Outliers, corresponding to multiply tethered beads (L_p_<28 nm) were removed (for a scatter plot see Fig. S12).

A number of effects, some intrinsic to the use of magnetic tweezers, contribute to the observed length dependence of L_p_. Seol et al. have shown that the effects of chain-end boundary conditions and bead fluctuations account well for the observed length-dependence of L_p_ measured in optical tweezers (OT) [28]. The magnitude of the length-dependence of the L_p_ observed in our MT experiments can however not be accounted for by these effects alone. In MT-based elastic-response measurements, bead-height fluctuations are limited by the stiffness of the molecule only and are not additionally restricted by a harmonic trap potential as in OT. Therefore the magnitude of bead height fluctuations in MT is larger than in OT measurements. Bead-wall steric exclusion effects accordingly have a greater effect [29]. Furthermore, the stiffness of the molecule and the effective viscosity experienced by the bead cannot readily be considered constant over the scale of the height fluctuations (see below).

To gain insight into the influence of the above effects on the MT-based measurements of the elastic properties of DNA, we have modeled the mechanical force response of a DNA bead-tether using a finite-difference-based approximation of the dynamic equation of motion. In the numerical simulations, the DNA length and stiffness were modeled using the 7-parameter analytical approximation to the WLC model proposed by Bouchiat et al. [30] (L_p_ = 50 nm). We furthermore have taken into account Faxén’s correction to the drag force for motion perpendicular to the wall [31]. And lastly, we introduced a Langevin noise force acting on the bead [1]. The simulated force responses were fit using the procedure outlined above. Fig. 5a shows L_p_ versus L_c_ predicted by the model in the absence (black line) and presence of thermal noise (blue line). In the absence of thermal noise, the resulting L_p_ is length-independent (L_p_ = 50 nm). In the presence of thermal noise, the modeled L_p_ values agree well with the experimental data and show a similar length-dependence. The discrepancy between actual and measured L_p_ observed in both the experiments and simulation is thus explained by the non-linear mechanical response to thermal fluctuations. For models in which all non-linear terms are excluded, the actual L_p_ is retrieved (data not shown). Our numerical simulations thus illustrate the importance of large-scale bead fluctuations on measurements of the elastic properties of DNA in MT experiments. In future work, we will further examine the consequences of the non-linear mechanical properties of a DNA-bead tether on measurements of elastic properties using MT.

Fig. 5b shows a histogram of the force-calibration factor measured for dsDNA molecules (N = 120, length 7.3 kb), normalized with respect to the mean, F_0_/<F_0_>. We find a standard deviation for the normalized force-calibration factor of σ_F0_ = 0.11, consistent with the previously reported bead-to-bead variation of 5–10% [20]. Next, we analyzed the distribution of attachment points for an ensemble of 7.3 kb dsDNA molecules (N = 45). The position of the DNA-attachment point on the surface of the bead was measured by analyzing the rotational response of the DNA-bead tether subject to a rotating external magnet pair as described above. Fig. 5c shows the extracted probability density of DNA-bead attachment offsets. The measured probability distribution closely matches the distribution that is expected based on the geometry and the rotational degree of freedom of the bead:


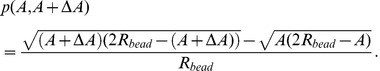
(3)

A derivation of eq. (3) can be found in the supplementary methods section (Methods S1).

### Conclusion

Here, we have systematically investigated the limits encountered when scaling MMT towards higher levels of parallelization, and we have provided a number of solutions to various issues encountered. We have implemented a post-experiment analysis of recorded images that provides a scalable solution to the computational challenge of parallel position detection of many beads (>100). Next, we present a model for the calculation of the forces exerted on paramagnetic beads in 3D. We furthermore have demonstrated that the angle of the force acting on DNA-bead tethers can be extracted experimentally from measurements of the rotational response of the DNA-bead tether. A thorough understanding and methods to characterize the distribution of force fields will make it possible to design MMT with larger fields of views, ultimately leading to a higher data-throughput in experiments. Finally, we have described methods and guidelines for the rapid characterization of the elastic properties of DNA-bead tethers and we used these methods to study the mechanical properties of DNA at the ensemble level.

Multiplexed magnetic tweezers will further benefit from ongoing developments in camera hardware that will make faster cameras with larger pixel arrays available to experimentalists. Elsewhere, we have demonstrated the ability to increase the density of beads in a field of view by means of targeted DNA-bead tethering to form dense, regular arrays [17]. Together with these developments, the guidelines and methods presented here will allow researchers to routinely set-up MMT measurements on many (>10^3^) molecules in parallel.

Such massively parallel MT allow single-experimental-run measurements of the statistical distribution of reaction rates and thereby greatly facilitate performing force- or torque spectroscopy analyses of DNA-enzyme interactions. MMT provide direct access to ensemble-level data while remaining sensitive to sample heterogeneity, providing a means to study infrequently occurring events in DNA-protein interactions.

## Supporting Information

Figure S1 **Offline image analysis and JPEG image compression. (a)** Example image of the field of view during an experiment. **(b)** Analysis of the compression quality level on the final tracking resolution. The tracking resolution in x, y and z is plotted for different JPEG compression levels (JPEG quality level 600 – 960, as defined by the Labview IMAQ software), as measured by analyzing the variance in the measured xyz position over 1000 frames for 8–10 beads that were rigidly attached to the flow cell slide. The solid blue line indicates the tracking resolution for uncompressed images. **(c)** JPEG compression versus time required for saving of the image to the hard drive. **(d)** Tracking resolution as function of image save time. For a JPEG quality factor >900, the tracking accuracy in z, σ_z_, asymptotes to σ_z_ = 10±1 nm. A JPEG quality level of 900 (corresponding to an image save time of 14 ms) and a 50 Hz acquisition rate were used throughout this work leading to a resolution in z, σ_z_ = 12±2 nm, and a resolution in x and y, σ_x,y_ = 2.2±0.5 nm. Simulations of the dynamic response of DNA-bead tethers were used to investigate the influence of the tracking resolution on the mechanical parameters extracted in experiments (see below). This analysis indicates that the tracking accuracy does not affect the extracted mechanical parameters using the analysis described for σ_z_ <30 nm (data not shown).
(TIF)

Figure S2
**Comparison of a measured (a) and simulated (b) response of a 7.3 kb dsDNA subject to a time-varying force (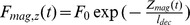, where F_0_ = 6.4 pN, l_dec_ = 1.55 mm and Z_mag_(t) = 1 mm + v_mag_t, with v_mag_ the speed of the magnet movement (v_mag_ = 0.1 mm/s)).** A good agreement between the simulated and measured force response is found. (c) Variance of the measured (red markers) and simulated (blue line) end-to-end distance versus the mean of the measured and simulated end-to-end distance respectively. Here, the DNA-bead tether was subject to a fixed force during a fixed time interval. In the simulation we have taken into account the camera-noise-induced error in determination of the length offset.(TIF)

Figure S3 **Drag force during a dynamic measurement of the force response of a DNA. (a)** Calculation of the magnitude of the drag force in the vertical direction acting on a 1 micron bead during measurement of the force response of a 7.3 kb DNA molecule using the procedure outlined in the main text. The magnet height is moved upward with a constant speed (0.1 mm/s), giving rise to an exponentially decreasing F_mag,z_. The model takes into account Faxén’s correction to the drag force due to the presence of a neighboring wall. The drag force is <0.6 fN throughout the experiment and can therefore be disregarded. **(b)** Height of bead as function of time during the experiment.
(TIF)

Figure S4 **Bead attachment offset and consequences for analysis of the DNA elastic properties.** (a) Force response of 7.3 kb dsDNA with bead attachment offset A = 0 (red line) and bead attachment offset A  =  R_bead_ = 0.5 µm (blue line). The bead attachment offset leads to an underestimation of the molecule’s end-to-end distance and an underestimation of the force exerted on the molecule, F_measured_  =  F_real_ (L_meas_/L_real_), where L_meas_ is the measured end-to-end distance and L_real_ is the real end-to-end distance. Accordingly, the persistence length extracted from the force response of the molecule is smaller than the real persistence length of the molecule. **(b)** Difference between the true and measured persistence length for a dsDNA molecule with bead attachment offset A  =  R_bead_. The graph illustrates the importance of an independent measurement of the bead attachment, particularly for short molecules.
(TIF)

Figure S5 **Schematic of bead indicating geometric parameters used to calculate the probability density function of DNA attachment offsets.** The bead has a preferred axis in paramagnetic polarizability that aligns along the direction of the magnetic field. The bead is free to rotate about the magnetic field axis. Molecules that bind to the segment of the sphere marked in red and defined by angles α and α + dα lead to a bead attachment offset in the range A to A + ΔA.
(TIF)

Figure S6 **3D modeling of magnetic fields and force.** (a) Schematic of the magnet configuration used in this work. The magnetic fields are calculated assuming a fictitious, uniformly distributed magnetic charge on the top and bottom surface of the magnets. The magnet gap size used in this work was 2 mm. (b) magnetic field distribution (blue line) compared to hall probe measurements on a magnet configuration with identical dimensions (circles data taken from ref [4]). (c) Force as function of magnet position calculated for the magnet configuration used in this work, calculated using the model described in this work (blue line) and final element simulations described in ref [4] (red line). (d–e) Force acting on a paramagnetic bead at different positions in a 400×400 µm field of view for a magnet height of 2 mm and 0 mm. A maximum force variation of 0.3% and 24% were found for a magnet height of 2 and 0 mm respectively.
(TIF)

Figure S7 **Force scaling factor and deviation from single exponential behavior. (a)** Vertical force acting on a paramagnetic bead aligned to the rotation axis of the magnet pair as function of magnet height in the range of magnet positions used in this work (Z_mag_>1 mm). The red line shows the best single exponential fit. **(b)** Relative error due to the single exponent assumption. **(c)** Absolute error related to this assumption. These graphs show that only a small error is introduced by assuming single exponential behavior and a related single force scaling factor, F_0_, for the dynamic force response measurement described in the main text.
(TIF)

Figure S8 **Comparison of Model and Measurement of Forces.** Vertical force acting on beads for different magnet heights. The blue line is given by finite element simulation while the red is measured data using 12 kb dsDNA averaged over 36 beads. Exponential fitting of the individual measured traces gives decay length, l_dec_, of 1.54 mm while simulation gives l_dec_ = 1.86 mm. A small discrepancy between model predictions and experiments was similarly observed by Lipfert et. al. (ref (4)) for a similar apparatus and different simulation methods. The applied force is a function of the magnetic field gradient, as well as the magnetic properties of the beads parameterized. A discrepancy between the vendor-quoted and actual values of B0 offers a possible explanation for the observed discrepancy in l_dec_. Results from 3D modeling however suggest that B_0_ must differ by a factor of ∼5 from the vendor-cited value to account for the observed l_dec_ discrepancy (data not shown). The l_dec_ discrepancy also could be caused by a misalignment of the magnet or a misevaluation of the inter-magnet gap-size. Simulations however indicate that magnet center position or inter-magnet gap size need to deviate drastically from expected values to offer an explanation (data not shown). A likely explanation is that the finite magnetic polarizability of the objective lens or flow-cell mount, which is not accounted for in the field simulation, change the magnetic field gradient in the vicinity of the bead.
(TIF)

Figure S9 **Effect of reorganizing data on scatter in energy versus extension plots.** (a) Dynamic force response measured for a 7.3 kb dsDNA with data points organized chronologically. (b) Same data, with the datapoints organized with ascending extension. (c) Energy versus extension calculated following eq. 2 in the main text. Large fluctuations in extension lead to scatter in the energy versus extension plot. The data is fit here by restricting the fit to the envelope of the energy versus extension curve. (d) Energy versus extension from data after re-ordering (as in panel b).
(TIF)

Figure S10 **Rotational response of the DNA-bead tether. (a)** Time-dependent response of DNA-bead tethers for the example of a bi-circular response (Z_mag_ = 0.2 mm, distance to the center = 0.2 mm R_att_ = 0.12 µm, R_prec_ = 0.25 µm) (bottom). The time-dependent angle of the rotating magnet is plotted on top. The time dependent response displays a single and double frequency contribution. This is due to an eccentric attachment point as well as the tether’s misalignment from the rotation axis of the magnet pair. **(b)** Time-dependent response of DNA-bead tethers for the example of a mono-circular response (Z_mag_ = 1 mm, d_center = _0.0 mm, R_att_ = 0.12 µm, R_prec_ = 0.02). In this case, the effect from the tether’s misalignment from the rotation axis of the magnets pair is small, leaving only a response from the eccentric attachment point.
(TIF)

Figure S11 **Radius of precession of a DNA-bead tether.** Precession radius measured for a DNA bead-tether subject to a rotating magnetic field as function of the distance of the tether to the rotation axis of the magnet pair and for different distances of the bottom of the magnet pair to the top of the flow cell (Z_mag_ = 0.2 mm, blue points, Z_mag_ = 0.5 mm, black points, Z_mag_ = 1 mm, red points). The data is compared to results from calculations using the above described formalism (Z_mag_ = 0.2 mm, blue line, Z_mag_ = 0.5 mm, black line, Z_mag_ = 1 mm, red line). A good agreement is found for Z_mag_ = 1 mm. The experimental data and model predictions deviate substantially for Z_mag_ <1 mm. The predicted and measured vertical force for DNA-bead tethers aligned to the rotation axis of the magnet pair were found to similarly deviate for Z_mag_<1 mm (The same observation was made in Ref. [4]). The discrepancy between model predictions and experimental data are possibly explained by a magnetization induced in the objective or metal parts of the flow cell holder that would change the magnetic field distribution when the magnet is placed close to the flow cell top.
(TIF)

Figure S12 **Scatter plot and outlier removal.** Ensemble analysis of DNA mechanics. **(a)** Measured persistence length, L_p_ and contour length, L_c_, for DNA molecules with different length (2.2 kb, 7.3 kb, 12 kb and 20 kb). The data is compared to predictions from finite difference modeling in the absence of thermal fluctuations (black line) and presence of thermal fluctuations (blue line). The data in the main text shows the mean of the measured distributions after removal of outliers that correspond to multiply tethered beads (i.e. L_p_<28 nm).
(TIF)

Methods S1 **Supplementary methods.** In this document we provide a detailed description of (1) the finite difference based model of the force-response of a DNA-bead tether, (2) a derivation of the probability density of DNA-bead attachment offsets (eq. 3), and (3) the formalism used for the calculation of the 3D force-field developed by the magnet pair.
(DOC)

Video S1 **Animation of the rotational response of a DNA-bead tether in the case of a misaligned magnet and centric DNA-attachment position.** The rotational response of a DNA-bead tether that is misaligned with respect to the axis of rotation of the magnet pair for the case where the DNA molecule is attached to the geometric bottom of the magnetic bead. The DNA-bead tether undergoes a precessional motion with a frequency equal to twice the frequency of the magnet rotation.
(GIF)

Video S2 **Animation of the rotational response of a DNA-bead tether in the case of an eccentric DNA-attachment position.** The rotational response of a DNA-bead tether that is aligned to the axis of rotation of the magnet pair and for which the DNA attachment position is eccentric. The DNA-bead tether undergoes a circular motion about its attachment point with a frequency that is equal to the frequency of the magnet rotation.
(GIF)

Video S3 **Animation of the rotational response of a DNA-bead tether in the case of a misaligned magnet and an eccentric DNA-attachment position.** The rotational response of a DNA-bead tether that is misaligned with respect to the axis of rotation of the magnet pair and for which the DNA attachment position is eccentric, is a combination of a precessional motion with ω_prec_ and a rotational motion ω_att_, where ω_prec_ = 2ω_att_. The combined, bi-circular motion gives rise to a rotational pattern in the plane parallel to the surface that is a special case of the epitrochoid: the Limaçon.
(GIF)
