# Supplementary figures and images for: Magnetic Forces and DNA Mechanics in Multiplexed Magnetic Tweezers
Source: PLoS One. 2012 Aug 3;7(8):e41432. doi: 10.1371/journal.pone.0041432 (PMC3411724; doi:10.1371/journal.pone.0041432)

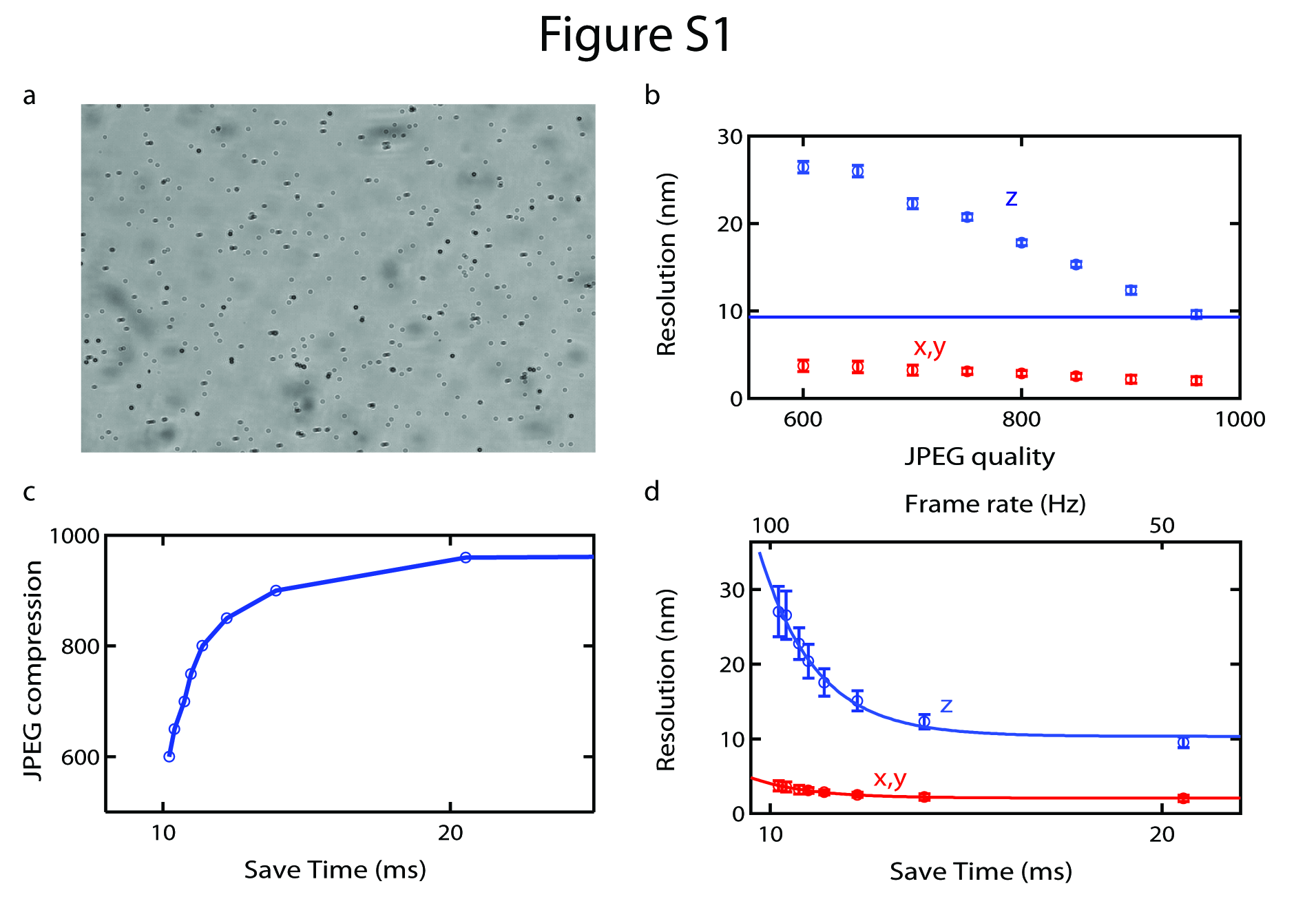

Supplement: Figure S1 — Offline image analysis and JPEG image compression. (a) Example image of the field of view during an experiment. (b) Analysis of the compression quality level on the final tracking resolution. The tracking resolution in x, y and z is plotted for different JPEG compression levels (JPEG quality level 600 – 960, as defined by the Labview IMAQ software), as measured by analyzing the variance in the measured xyz position over 1000 frames for 8–10 beads that were rigidly attached to the flow cell slide. The solid blue line indicates the tracking resolution for uncompressed images. (c) JPEG compression versus time required for saving of the image to the hard drive. (d) Tracking resolution as function of image save time. For a JPEG quality factor >900, the tracking accuracy in z, σz, asymptotes to σz = 10±1 nm. A JPEG quality level of 900 (corresponding to an image save time of 14 ms) and a 50 Hz acquisition rate were used throughout this work leading to a resolution in z, σz = 12±2 nm, and a resolution in x and y, σx,y = 2.2±0.5 nm. Simulations of the dynamic response of DNA-bead tethers were used to investigate the influence of the tracking resolution on the mechanical parameters extracted in experiments (see below). This analysis indicates that the tracking accuracy does not affect the extracted mechanical parameters using the analysis described for σz <30 nm (data not shown). (TIF) [file pone.0041432.s001.tif]

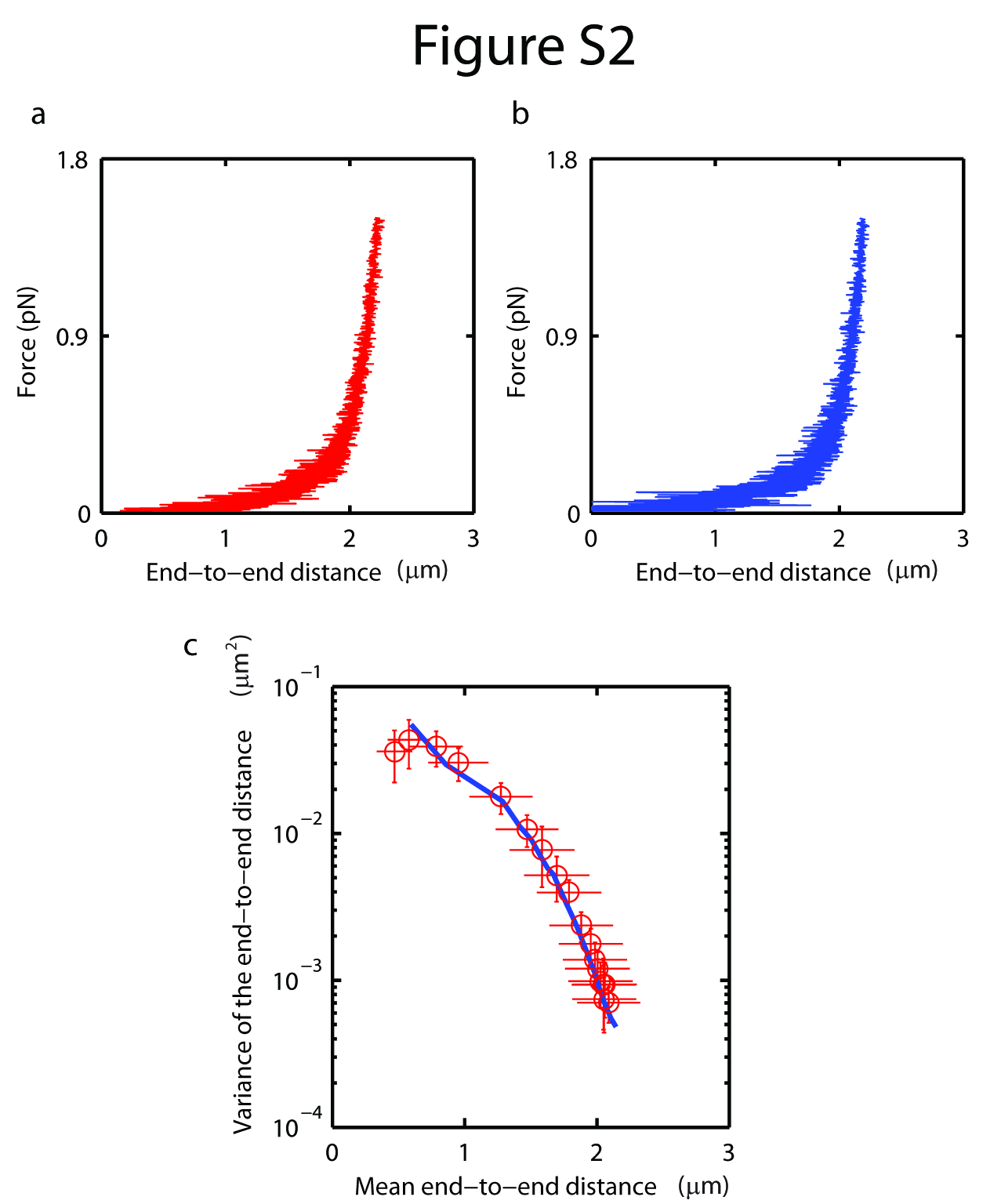

Supplement: Figure S2 — Comparison of a measured (a) and simulated (b) response of a 7.3 kb dsDNA subject to a time-varying force (, where F0 = 6.4 pN, ldec = 1.55 mm and Zmag(t) = 1 mm + vmagt, with vmag the speed of the magnet movement (vmag = 0.1 mm/s)). A good agreement between the simulated and measured force response is found. (c) Variance of the measured (red markers) and simulated (blue line) end-to-end distance versus the mean of the measured and simulated end-to-end distance respectively. Here, the DNA-bead tether was subject to a fixed force during a fixed time interval. In the simulation we have taken into account the camera-noise-induced error in determination of the length offset. (TIF) [file pone.0041432.s002.tif]

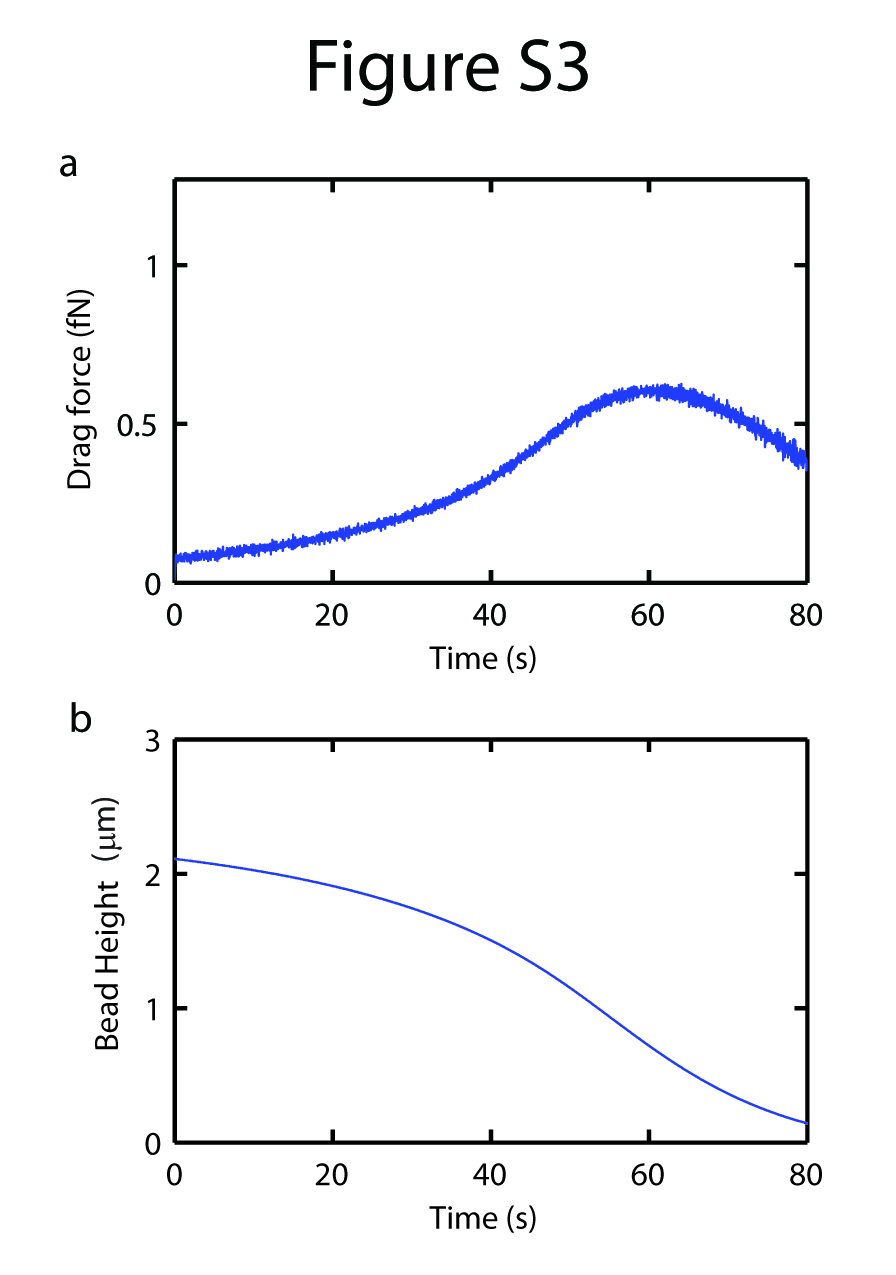

Supplement: Figure S3 — Drag force during a dynamic measurement of the force response of a DNA. (a) Calculation of the magnitude of the drag force in the vertical direction acting on a 1 micron bead during measurement of the force response of a 7.3 kb DNA molecule using the procedure outlined in the main text. The magnet height is moved upward with a constant speed (0.1 mm/s), giving rise to an exponentially decreasing Fmag,z. The model takes into account Faxén’s correction to the drag force due to the presence of a neighboring wall. The drag force is <0.6 fN throughout the experiment and can therefore be disregarded. (b) Height of bead as function of time during the experiment. (TIF) [file pone.0041432.s003.tif]

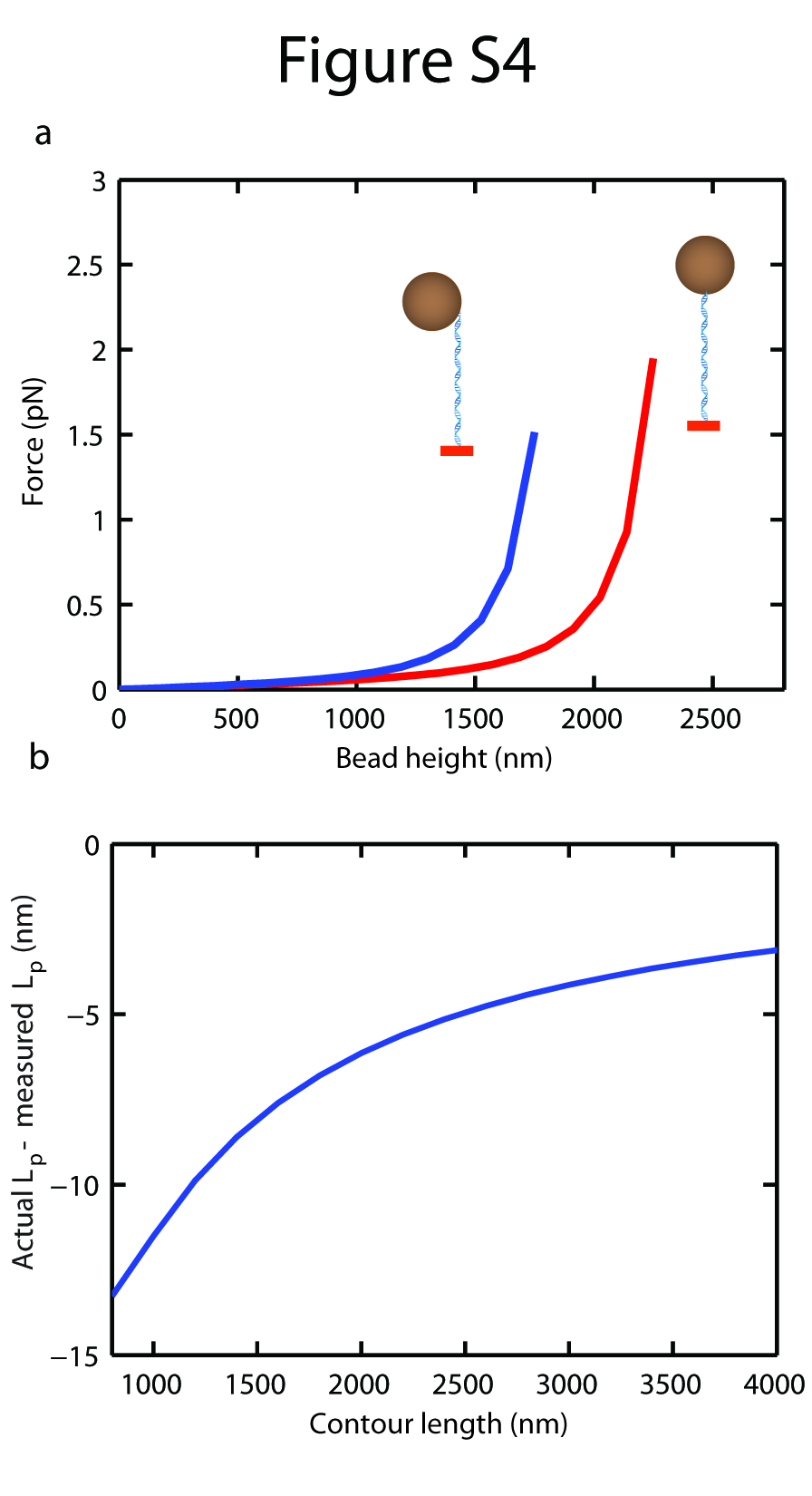

Supplement: Figure S4 — Bead attachment offset and consequences for analysis of the DNA elastic properties. (a) Force response of 7.3 kb dsDNA with bead attachment offset A = 0 (red line) and bead attachment offset A = Rbead = 0.5 µm (blue line). The bead attachment offset leads to an underestimation of the molecule’s end-to-end distance and an underestimation of the force exerted on the molecule, Fmeasured = Freal (Lmeas/Lreal), where Lmeas is the measured end-to-end distance and Lreal is the real end-to-end distance. Accordingly, the persistence length extracted from the force response of the molecule is smaller than the real persistence length of the molecule. (b) Difference between the true and measured persistence length for a dsDNA molecule with bead attachment offset A = Rbead. The graph illustrates the importance of an independent measurement of the bead attachment, particularly for short molecules. (TIF) [file pone.0041432.s004.tif]

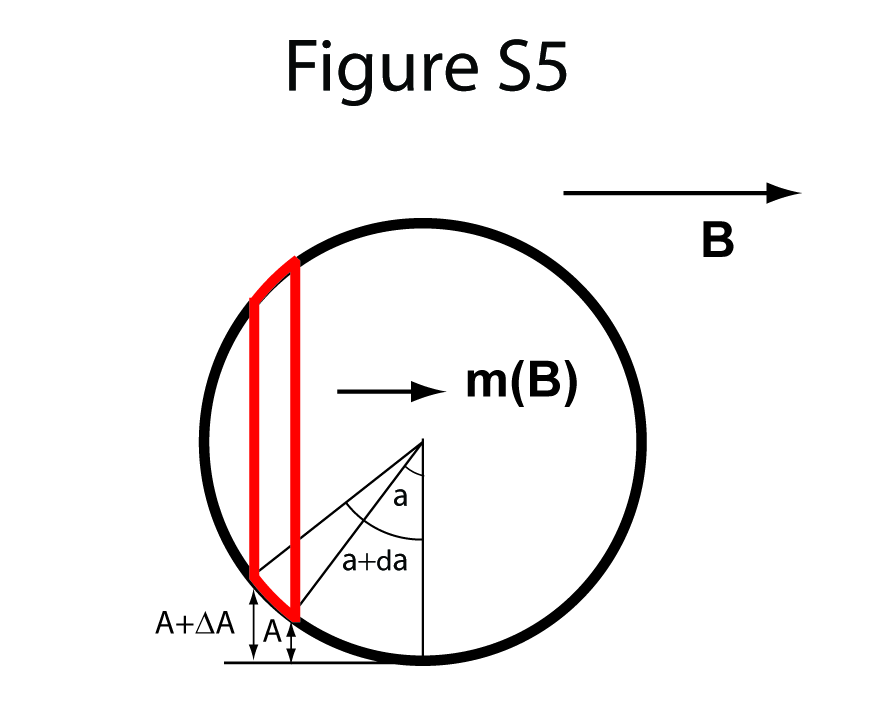

Supplement: Figure S5 — Schematic of bead indicating geometric parameters used to calculate the probability density function of DNA attachment offsets. The bead has a preferred axis in paramagnetic polarizability that aligns along the direction of the magnetic field. The bead is free to rotate about the magnetic field axis. Molecules that bind to the segment of the sphere marked in red and defined by angles α and α + dα lead to a bead attachment offset in the range A to A + ΔA. (TIF) [file pone.0041432.s005.tif]

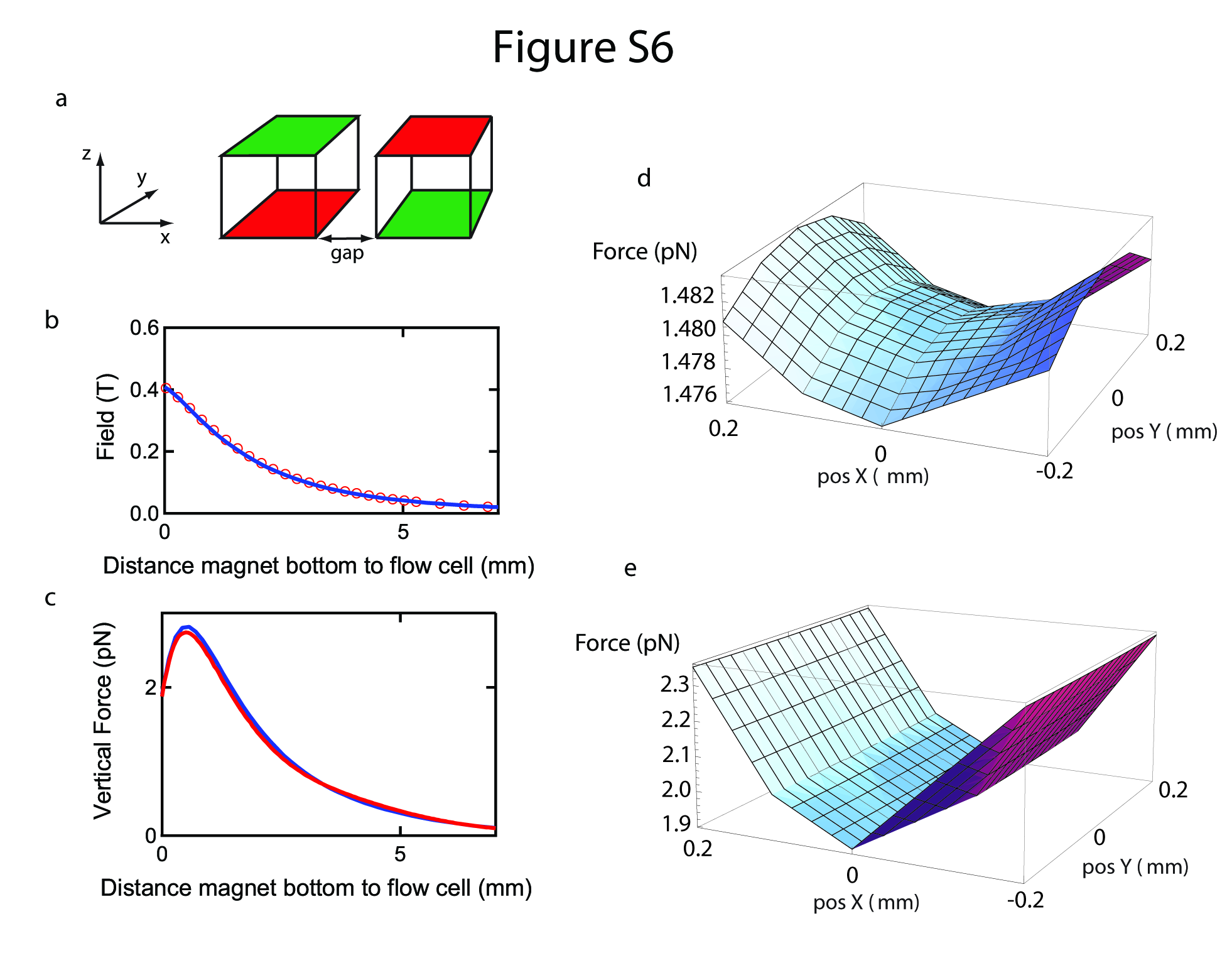

Supplement: Figure S6 — 3D modeling of magnetic fields and force. (a) Schematic of the magnet configuration used in this work. The magnetic fields are calculated assuming a fictitious, uniformly distributed magnetic charge on the top and bottom surface of the magnets. The magnet gap size used in this work was 2 mm. (b) magnetic field distribution (blue line) compared to hall probe measurements on a magnet configuration with identical dimensions (circles data taken from ref [4]). (c) Force as function of magnet position calculated for the magnet configuration used in this work, calculated using the model described in this work (blue line) and final element simulations described in ref [4] (red line). (d–e) Force acting on a paramagnetic bead at different positions in a 400×400 µm field of view for a magnet height of 2 mm and 0 mm. A maximum force variation of 0.3% and 24% were found for a magnet height of 2 and 0 mm respectively. (TIF) [file pone.0041432.s006.tif]

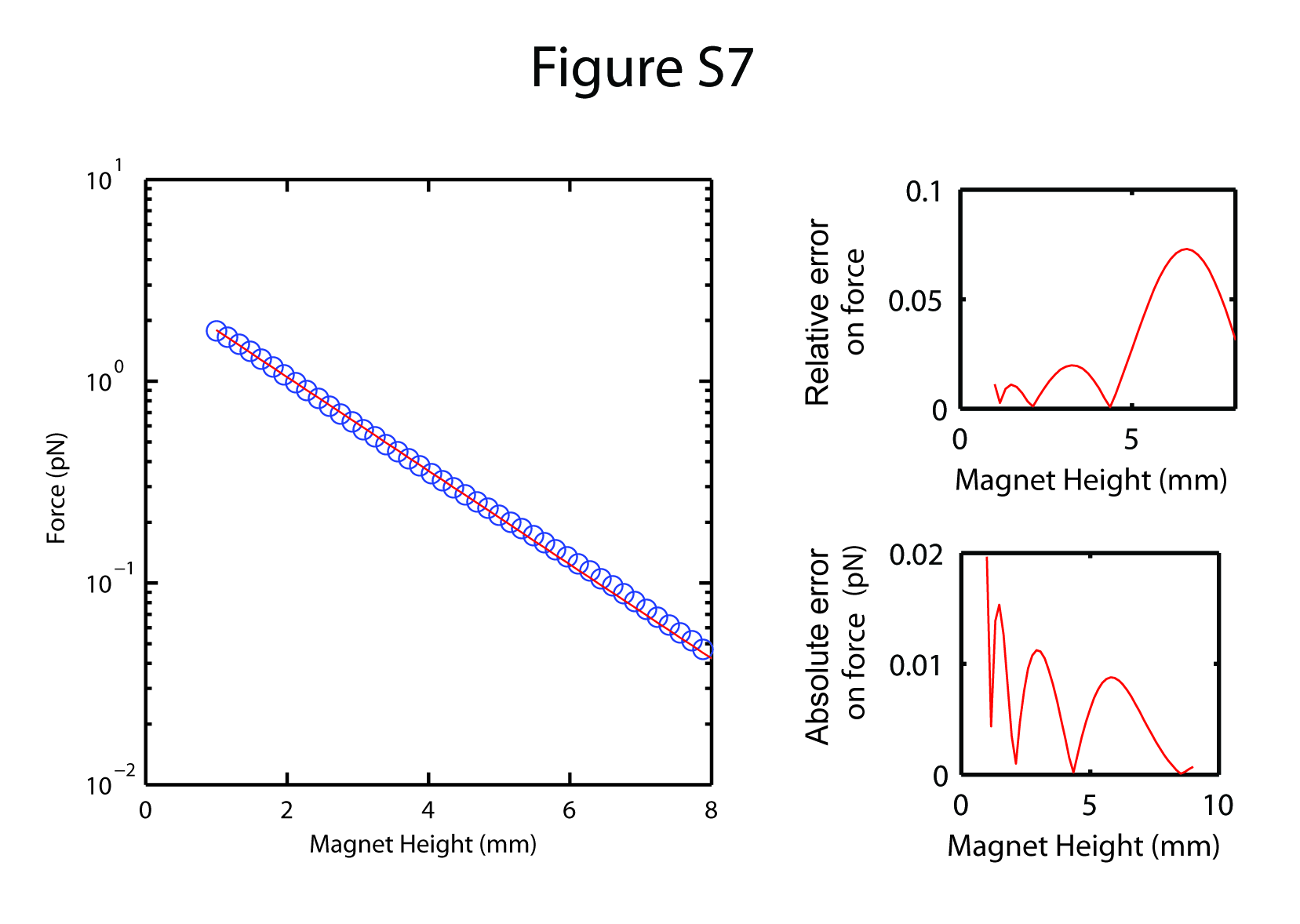

Supplement: Figure S7 — Force scaling factor and deviation from single exponential behavior. (a) Vertical force acting on a paramagnetic bead aligned to the rotation axis of the magnet pair as function of magnet height in the range of magnet positions used in this work (Zmag>1 mm). The red line shows the best single exponential fit. (b) Relative error due to the single exponent assumption. (c) Absolute error related to this assumption. These graphs show that only a small error is introduced by assuming single exponential behavior and a related single force scaling factor, F0, for the dynamic force response measurement described in the main text. (TIF) [file pone.0041432.s007.tif]

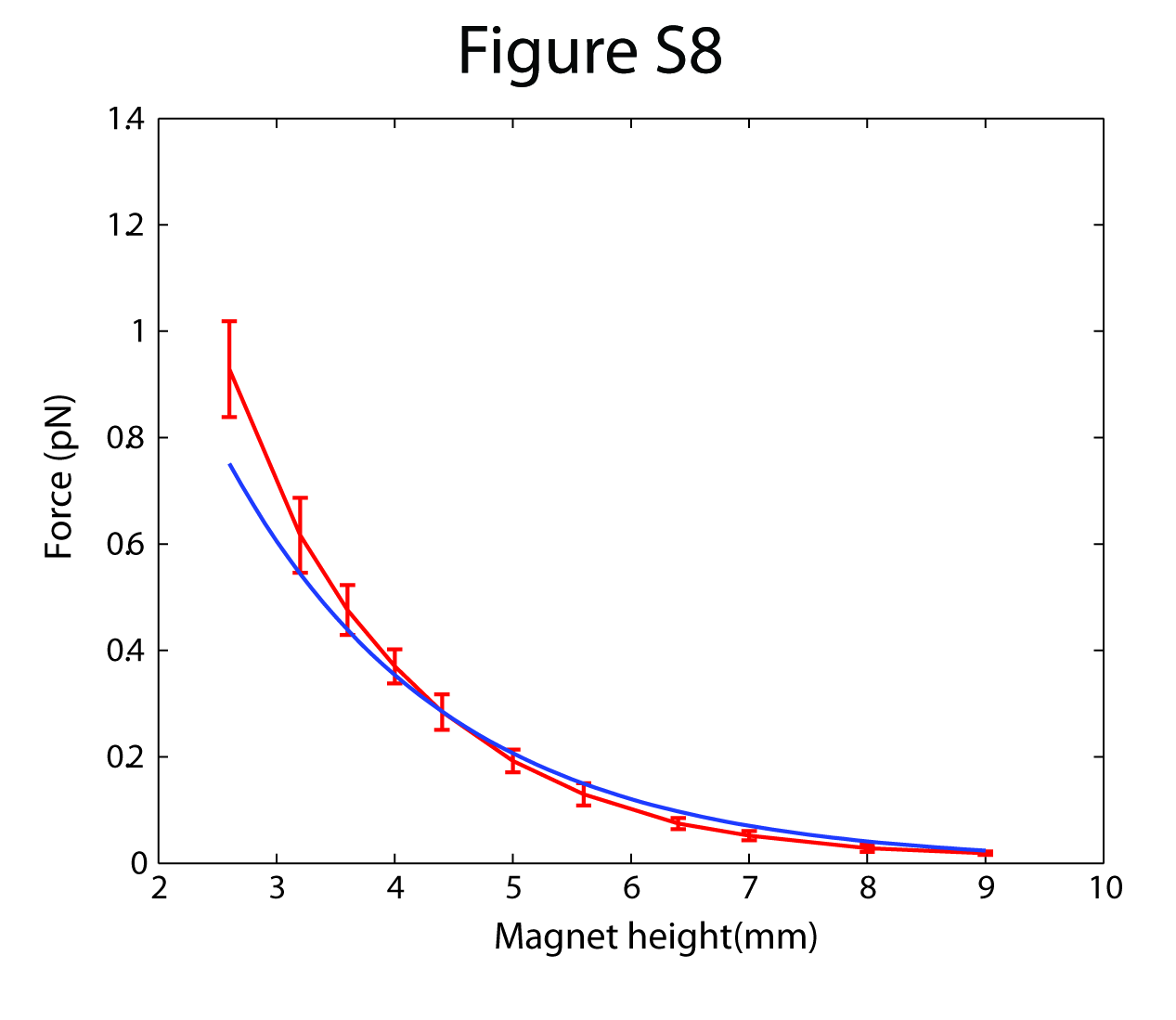

Supplement: Figure S8 — Comparison of Model and Measurement of Forces. Vertical force acting on beads for different magnet heights. The blue line is given by finite element simulation while the red is measured data using 12 kb dsDNA averaged over 36 beads. Exponential fitting of the individual measured traces gives decay length, ldec, of 1.54 mm while simulation gives ldec = 1.86 mm. A small discrepancy between model predictions and experiments was similarly observed by Lipfert et. al. (ref (4)) for a similar apparatus and different simulation methods. The applied force is a function of the magnetic field gradient, as well as the magnetic properties of the beads parameterized. A discrepancy between the vendor-quoted and actual values of B0 offers a possible explanation for the observed discrepancy in ldec. Results from 3D modeling however suggest that B0 must differ by a factor of ∼5 from the vendor-cited value to account for the observed ldec discrepancy (data not shown). The ldec discrepancy also could be caused by a misalignment of the magnet or a misevaluation of the inter-magnet gap-size. Simulations however indicate that magnet center position or inter-magnet gap size need to deviate drastically from expected values to offer an explanation (data not shown). A likely explanation is that the finite magnetic polarizability of the objective lens or flow-cell mount, which is not accounted for in the field simulation, change the magnetic field gradient in the vicinity of the bead. (TIF) [file pone.0041432.s008.tif]

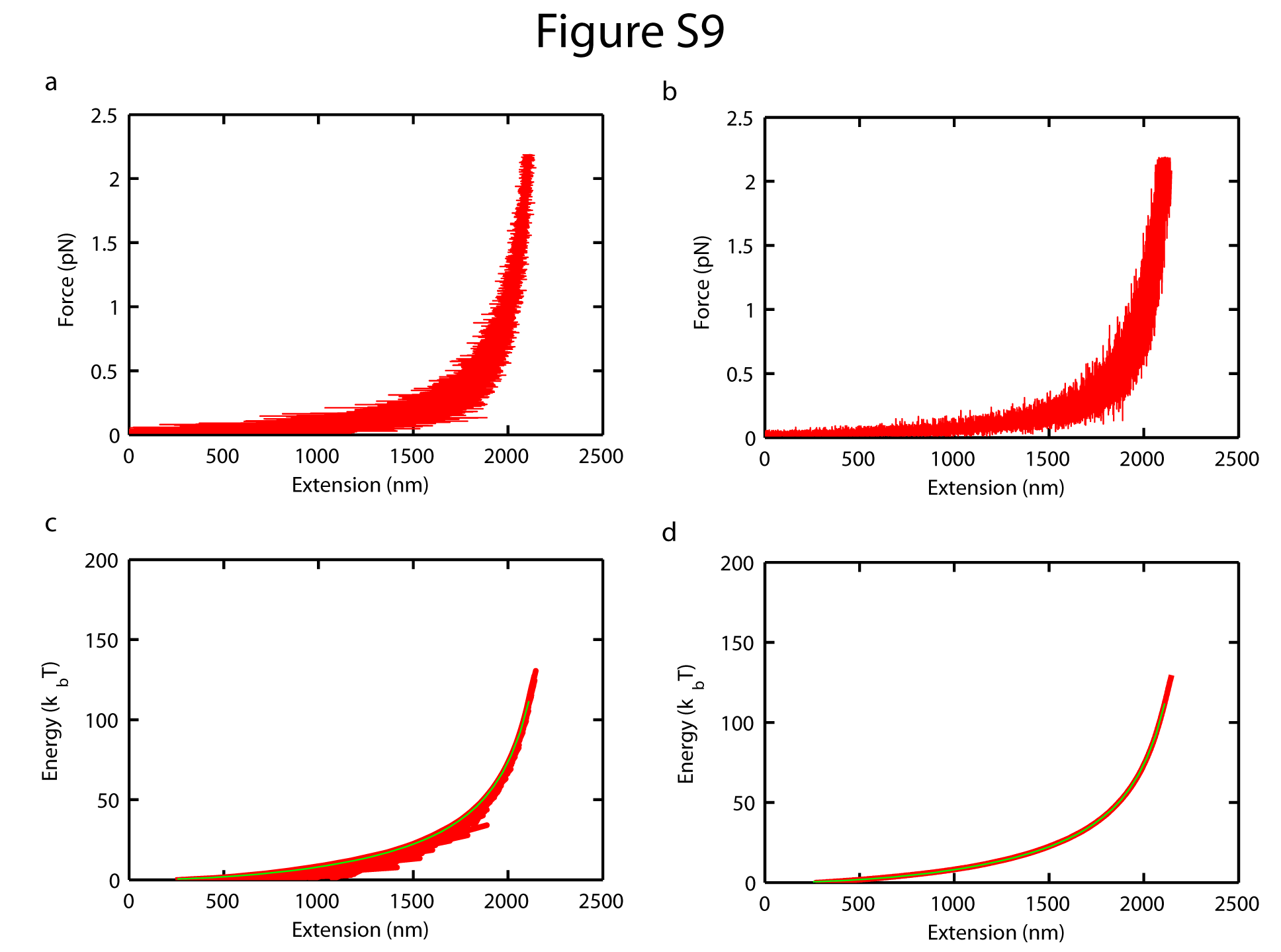

Supplement: Figure S9 — Effect of reorganizing data on scatter in energy versus extension plots. (a) Dynamic force response measured for a 7.3 kb dsDNA with data points organized chronologically. (b) Same data, with the datapoints organized with ascending extension. (c) Energy versus extension calculated following eq. 2 in the main text. Large fluctuations in extension lead to scatter in the energy versus extension plot. The data is fit here by restricting the fit to the envelope of the energy versus extension curve. (d) Energy versus extension from data after re-ordering (as in panel b). (TIF) [file pone.0041432.s009.tif]

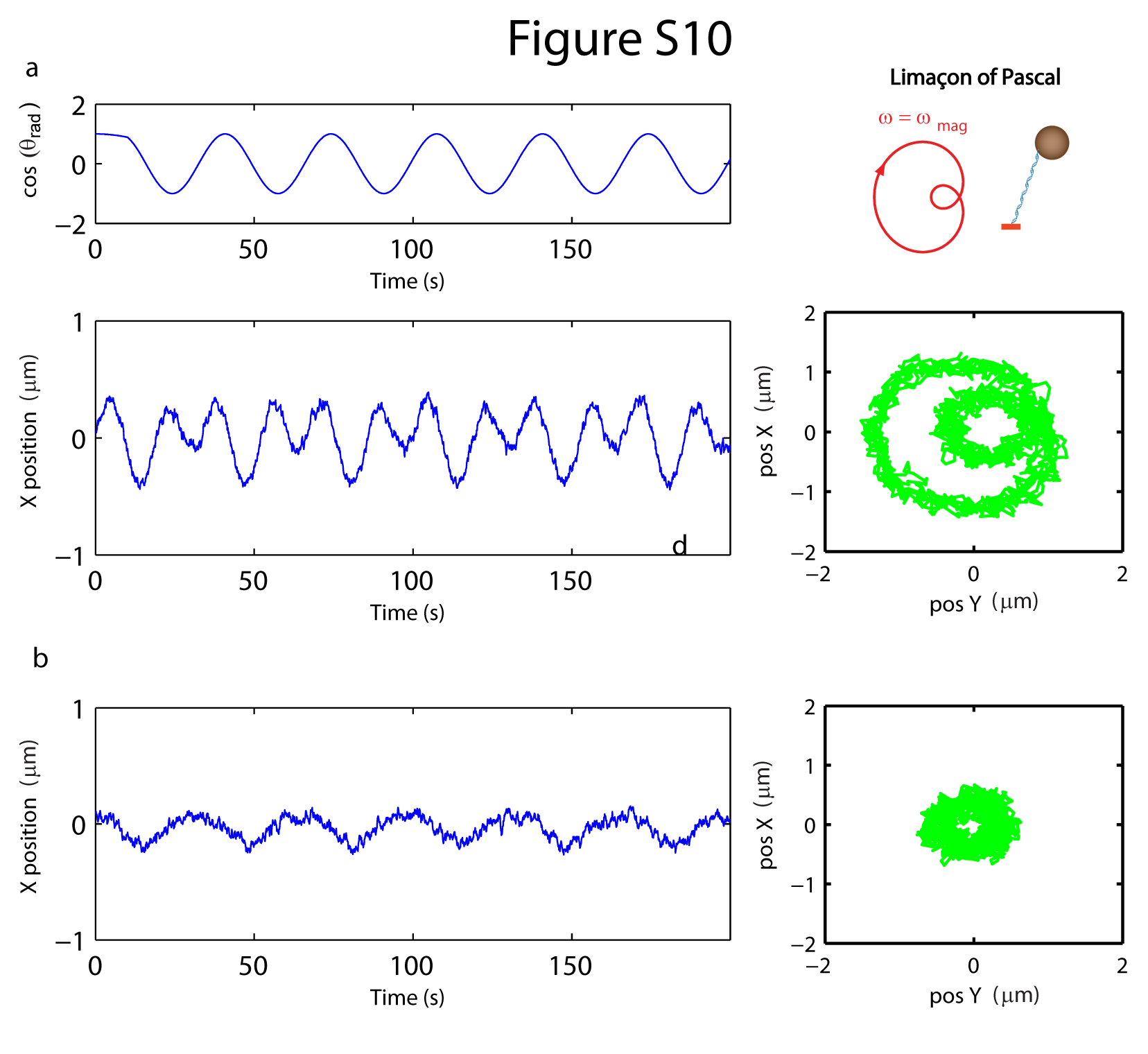

Supplement: Figure S10 — Rotational response of the DNA-bead tether. (a) Time-dependent response of DNA-bead tethers for the example of a bi-circular response (Zmag = 0.2 mm, distance to the center = 0.2 mm Ratt = 0.12 µm, Rprec = 0.25 µm) (bottom). The time-dependent angle of the rotating magnet is plotted on top. The time dependent response displays a single and double frequency contribution. This is due to an eccentric attachment point as well as the tether’s misalignment from the rotation axis of the magnet pair. (b) Time-dependent response of DNA-bead tethers for the example of a mono-circular response (Zmag = 1 mm, dcenter = 0.0 mm, Ratt = 0.12 µm, Rprec = 0.02). In this case, the effect from the tether’s misalignment from the rotation axis of the magnets pair is small, leaving only a response from the eccentric attachment point. (TIF) [file pone.0041432.s010.tif]

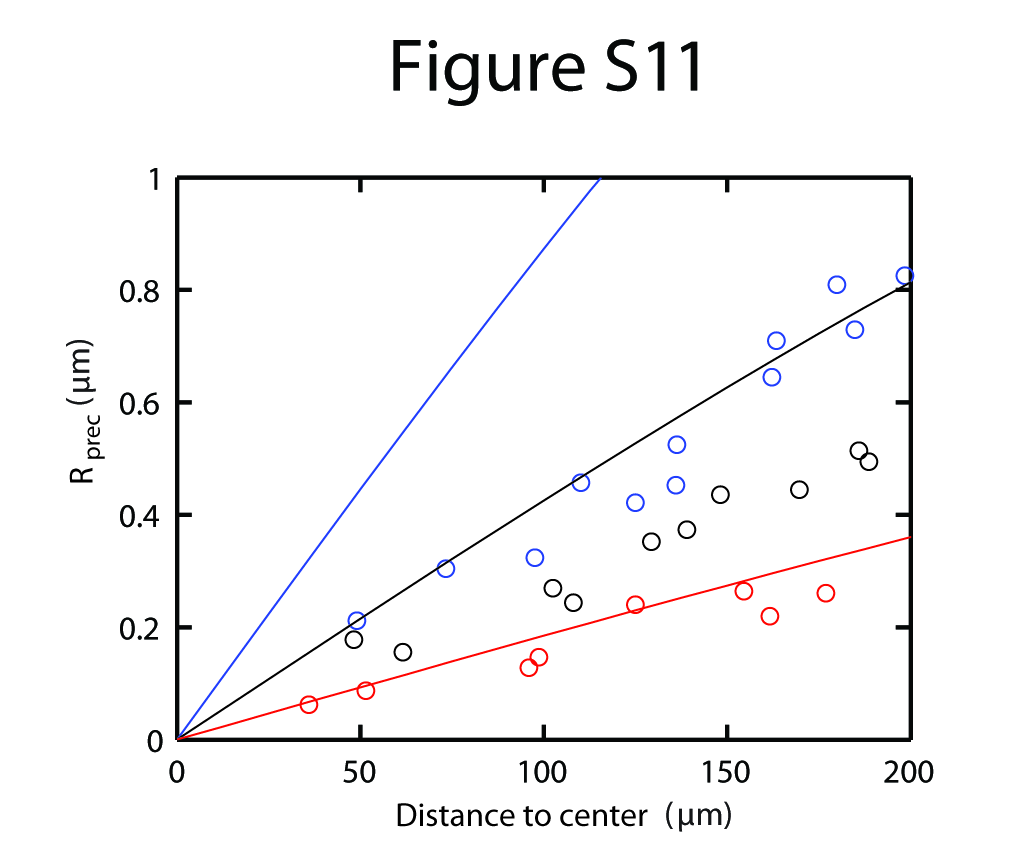

Supplement: Figure S11 — Radius of precession of a DNA-bead tether. Precession radius measured for a DNA bead-tether subject to a rotating magnetic field as function of the distance of the tether to the rotation axis of the magnet pair and for different distances of the bottom of the magnet pair to the top of the flow cell (Zmag = 0.2 mm, blue points, Zmag = 0.5 mm, black points, Zmag = 1 mm, red points). The data is compared to results from calculations using the above described formalism (Zmag = 0.2 mm, blue line, Zmag = 0.5 mm, black line, Zmag = 1 mm, red line). A good agreement is found for Zmag = 1 mm. The experimental data and model predictions deviate substantially for Zmag <1 mm. The predicted and measured vertical force for DNA-bead tethers aligned to the rotation axis of the magnet pair were found to similarly deviate for Zmag<1 mm (The same observation was made in Ref. [4]). The discrepancy between model predictions and experimental data are possibly explained by a magnetization induced in the objective or metal parts of the flow cell holder that would change the magnetic field distribution when the magnet is placed close to the flow cell top. (TIF) [file pone.0041432.s011.tif]

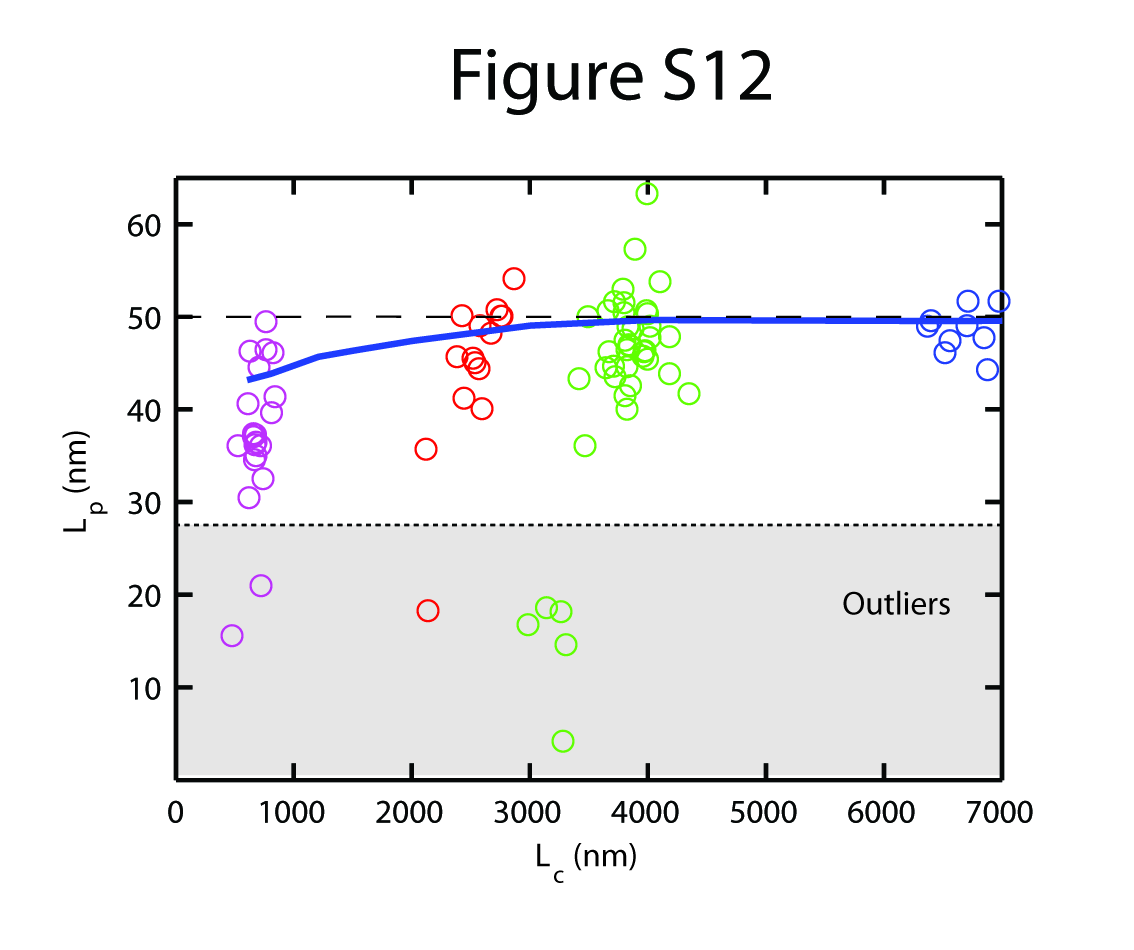

Supplement: Figure S12 — Scatter plot and outlier removal. Ensemble analysis of DNA mechanics. (a) Measured persistence length, Lp and contour length, Lc, for DNA molecules with different length (2.2 kb, 7.3 kb, 12 kb and 20 kb). The data is compared to predictions from finite difference modeling in the absence of thermal fluctuations (black line) and presence of thermal fluctuations (blue line). The data in the main text shows the mean of the measured distributions after removal of outliers that correspond to multiply tethered beads (i.e. Lp<28 nm). (TIF) [file pone.0041432.s012.tif]

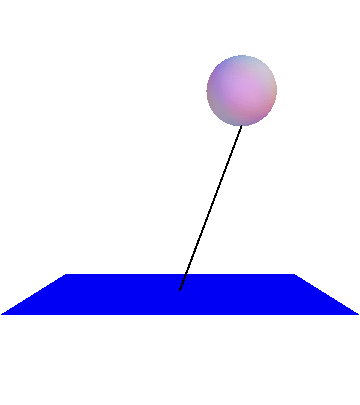

Supplement: Video S1 — Animation of the rotational response of a DNA-bead tether in the case of a misaligned magnet and centric DNA-attachment position. The rotational response of a DNA-bead tether that is misaligned with respect to the axis of rotation of the magnet pair for the case where the DNA molecule is attached to the geometric bottom of the magnetic bead. The DNA-bead tether undergoes a precessional motion with a frequency equal to twice the frequency of the magnet rotation. (GIF) [file pone.0041432.s014.gif]

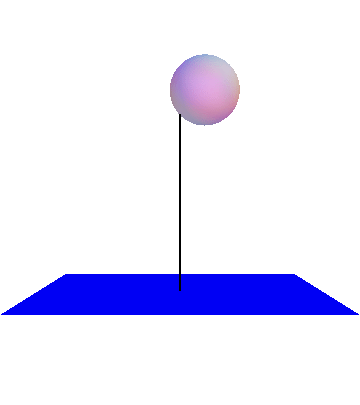

Supplement: Video S2 — Animation of the rotational response of a DNA-bead tether in the case of an eccentric DNA-attachment position. The rotational response of a DNA-bead tether that is aligned to the axis of rotation of the magnet pair and for which the DNA attachment position is eccentric. The DNA-bead tether undergoes a circular motion about its attachment point with a frequency that is equal to the frequency of the magnet rotation. (GIF) [file pone.0041432.s015.gif]

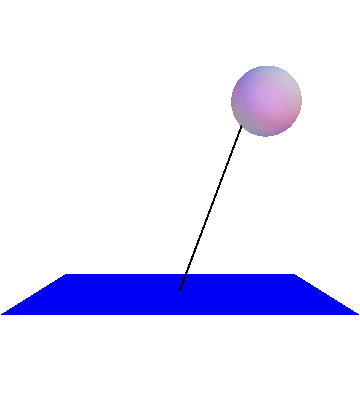

Supplement: Video S3 — Animation of the rotational response of a DNA-bead tether in the case of a misaligned magnet and an eccentric DNA-attachment position. The rotational response of a DNA-bead tether that is misaligned with respect to the axis of rotation of the magnet pair and for which the DNA attachment position is eccentric, is a combination of a precessional motion with ωprec and a rotational motion ωatt, where ωprec = 2ωatt. The combined, bi-circular motion gives rise to a rotational pattern in the plane parallel to the surface that is a special case of the epitrochoid: the Limaçon. (GIF) [file pone.0041432.s016.gif]
