# Supplementary material for: Magnetic Forces and DNA Mechanics in Multiplexed Magnetic Tweezers
Source: PLoS One. 2012 Aug 3;7(8):e41432. doi: 10.1371/journal.pone.0041432 (PMC3411724; doi:10.1371/journal.pone.0041432)
Supplement: Methods S1 — Supplementary methods. In this document we provide a detailed description of (1) the finite difference based model of the force-response of a DNA-bead tether, (2) a derivation of the probability density of DNA-bead attachment offsets (eq. 3), and (3) the formalism used for the calculation of the 3D force-field developed by the magnet pair. (DOC) [file pone.0041432.s013.doc]

Supplemental methods for:

# Magnetic forces and DNA mechanics in multiplexed magnetic tweezers

Iwijn De Vlaminck*, Thomas Henighan*, Marijn T.J. van Loenhout, Daniel Burnham, Cees Dekker

[Magnetic forces and DNA mechanics in multiplexed magnetic tweezers 1](#__RefHeading___Toc329799544)

[Supplementary methods 3](#__RefHeading___Toc329799545)

[Dynamic simulations of DNA-bead tethers using finite difference method 3](#__RefHeading___Toc329799546)

[Distribution of DNA attachment points 7](#__RefHeading___Toc329799547)

[3D modeling of force fields in magnetic tweezers 8](#__RefHeading___Toc329799548)

[Formalism and parameters 8](#__RefHeading___Toc329799549)

[Supporting References 11](#__RefHeading___Toc329799550)

# Supplementary methods

## Dynamic simulations of DNA-bead tethers using finite difference method

We have investigated the response of a DNA-bead tether to a time-dependent applied magnetic force using numerical simulations. The model is based on a finite difference approximation of the dynamic equation of motion of a DNA-bead tether and was implemented in MATLAB (The MathWorks, Natick, MA). The model provides insight in the magnitude of the drag force during dynamic force spectroscopy experiments and allows investigating the influence of the force response fitting algorithms used to extract values for the persistence length and contour lengths of the molecule. We furthermore used this modeling to investigate various factors that influence the force response analysis: (1) thermal force noise acting on the paramagnetic bead, (2) noise due to video-microscopy-based tracking of the z-position of the bead, (3) the effects of eccentric attachment of DNA to the magnetic bead, and (4) a position dependent effective viscosity due to wall effects.

The response of a bead-DNA tether subject to an applied magnetic force, Fmag,z, and thermal noise force, Fn, is well-described by following equation of motion (motion in z direction):

$6\pi\eta_{eff}R_{bead}\frac{dz(t)}{dt}+\frac{dF_{WLC}}{dz}dz(t)+F_{WLC}(z(t))=F_{mag,z}(t)+F_{n}(t)$. (1)

where Rbead is the bead radius, ηeff is the effective viscosity of the medium, and FWLC  is the force required to extend the molecule to a given extension z (see below).

The differential equation is numerically solved using a finite difference representation, where the extension at simulation step i+1, is calculated on the basis of the extension at step i, with a time step dt = 10-4 s:

$z(i+1)=z(i)+\frac{(F_{mag,z}(i)+F_{n}(i)-F_{WLC}z(i))dt}{(6\pi\eta_{eff}(z(i))R+dt\frac{dF_{WLC}(z(i))}{dz(i)})}$. (2)

The Langevin force noise acting on a 1 μm bead at room temperature, T=293 K, in a medium with viscosity η = 0.88 10-3 Ns/m2 can be calculated as:

$F_{n}=\sqrt{\frac{12\pi\text{k}_{\text{B}}\text{T}\eta R}{dt}}$, (3)

where, kb is the Boltzmann constant. The drag force acting on a bead translating in a fluid medium increases by the presence of a neighboring wall. In the numerical simulations, we have taken into account Faxén’s correction to the drag force for motion perpendicular to the wall. The position dependence of the effective viscosity using this correction is described by :

$\eta_{eff}(z(i))=\frac{\eta}{(1-\frac{9}{8}\frac{R}{z(i)}+\frac{1}{2}(\frac{R}{z(i)})^{3})}$ (4)

The mechanical properties of the DNA are linearly approximated. The force experienced by the bead is calculated using $F(z+dz)=F_{WLC}(z)+\frac{dF_{WLC}(z)}{dz}dz$, where FWLC­(z)is the force versus extension characteristic of dsDNA as described by the worm-like chain model , and $\frac{dF_{WLC}(z)}{dz}$is the slope of the force-distance curve at an extension z.

$F_{WLC}(z)=\frac{k_{b}T}{L_{p}}\left[\frac{1}{4(1-z/L_{0})^{2}}-\frac{1}{4}+\frac{z}{L_{0}}+\sum_{i=2}^{i\leq7} a_{i}(\frac{z}{L_{0}})^{i}\right],$ (5)

with *a*2= - 0.5164228, *a*3 = - 2.737418, *a*4 =16.07497, *a*5 =-38.87607, *a*6 = 39.49944, and *a*7 =-14.17718.

We have furthermore taken into account the experimental noise introduced by the errors made during position tracking, by adding Gaussian distributed position noise with standard deviation = 10 nm. Lastly, we have taken into account a camera frame rate of 50 Hz and a 2 ms camera shutter time as in the experiment.

We have used this model to simulate the response of a DNA-bead tether (7.3 kb dsDNA) subject to a time-varying force. Fig. S2 compares the force response measured in an experiment and a simulated force response for a DNA-bead tether subject to an applied force $F_{mag,z}(t)=F_{0}\exp(-\frac{Z_{mag}(t)}{l_{dec}})$, where F0 = 6.4 pN, ldec = 1.55 mm and Zmag(t) = 1 mm + vmagt, with vmag the speed of the magnet movement (vmag = 0.1 mm/s).

Next, we measured the variance and mean of the end-to-end distance of the tether at a constant force and compared to results from simulations. Fig. S2c shows the variance of the end-to-end distance as function of the mean of the end-to-end distance obtained in both simulations and experiments. The experimental data was obtained for N = 13 molecules and the error bars are the standard deviation. The plot shows a good agreement between the measured and simulated response of DNA-bead tether subject to a constant force. The effective stiffness of dsDNA is a strong function of the applied force, where the effective stiffness, $\frac{dF_{WLC}(z)}{dz}$, is lower at lower applied force (and corresponding smaller end-to-end distance). The variance of the measured end-to-end distance thus increases with decreasing force.

**
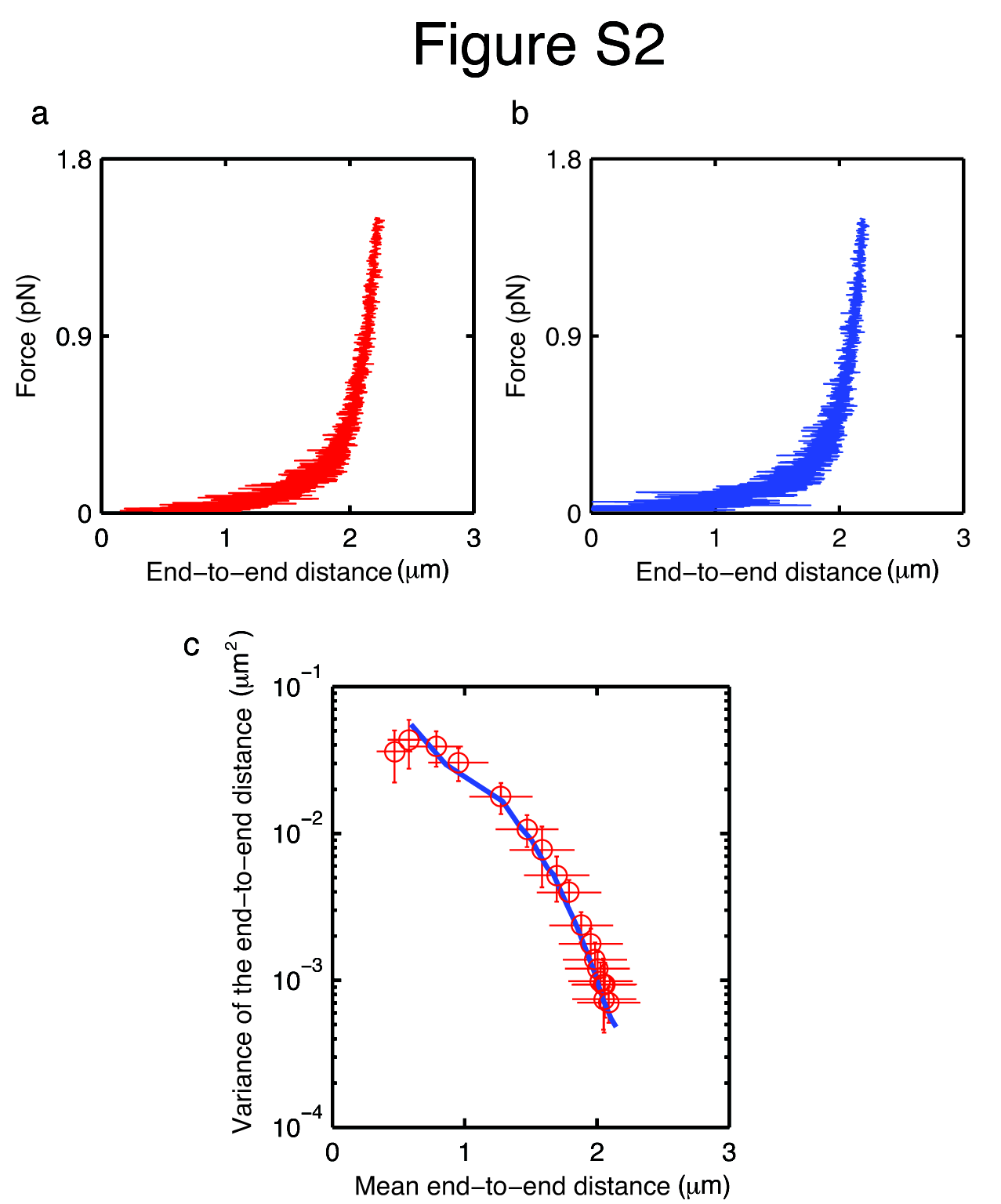
­**

**Figure S2.** Comparison of a measured (a) and simulated (b) response of a 7.3 kb dsDNA subject to a time-varying force ($F_{mag,z}(t)=F_{0}\exp(-\frac{Z_{mag}(t)}{l_{dec}})$, where F0 = 6.4 pN, ldec = 1.55 mm and Zmag(t) = 1 mm + vmagt, with vmag the speed of the magnet movement (vmag = 0.1 mm/s).). A good agreement between the simulated and measured force response is found. (c) Variance of the measured (red markers) and simulated (blue line) end-to-end distance versus the mean of the measured and simulated end-to-end distance respectively. Here, the DNA-bead tether was subject to a fixed force during a fixed time interval. In the simulation we have taken into account the camera-noise-induced error in determination of the length offset.

## Distribution of DNA attachment points

We derive the likelihood that a DNA-bead-tether displays a bead DNA attachment offset in the range A < A0 < A+ΔA. For this purpose, it is important to first consider the rotational degrees of freedom of the DNA-bead tether. As mentioned in the main text, the magnetic bead has a non-uniform paramagnetic polarizability . In the presence of a magnetic field, the paramagnetic bead experiences a torque that aligns the easy axis of the paramagnetic polarizability along the direction of the magnetic field. Any rotational motion of the bead around both axes that are perpendicular to the magnetic field axis is thus suppressed. The bead is however free to rotate around the axis parallel to the magnetic field. Upon application of a magnetic field, the applied force exerts a torque on the bead that re-orients the bead such that the DNA-bead attachment moves to a position along the bottom middle line of the bead. Attachment of the DNA in the absence of force on a position along the segmental surface area of the spherical bead defined by the angles α and α + dα, as indicated in Fig. S5, leads to a bead attachment offset in the range A to A + ΔA in the presence of a magnetic field and force. The probability density is then proportional to the relative area of the segment of the sphere surface to the total area of the sphere (see Fig. S5):

$p(A|A+\Delta A)=\frac{\Delta A_{segment}}{A_{sphere}}$. (6)

The segmental area can be calculated following:

$\Delta A_{segment}=\int_{A}^{A+\Delta A} (2\pi(R_{bead}-A))R_{bead}\frac{dA}{\sqrt{R_{bead}^{2}-(R_{bead}-A)^{2}}}$. (7)

Where Rbead is the radius of the bead. The probability function can thus be calculated as:

$p(A|A+\Delta A)=\int_{A}^{A+\Delta A} \frac{(R_{bead}-A)dA}{R_{bead}\sqrt{R_{bead}^{2}-(R_{bead}-A)^{2}}}=\frac{\sqrt{\left(A+\Delta A\right)(2R_{bead}-\left(A+\Delta A)\right)}-\sqrt{A(2R_{bead}-A)}}{R_{bead}}$ (8)

Figure 5c in the main text shows an excellent agreement between the measured probability distribution and the probability density expected from the geometric arguments presented here.


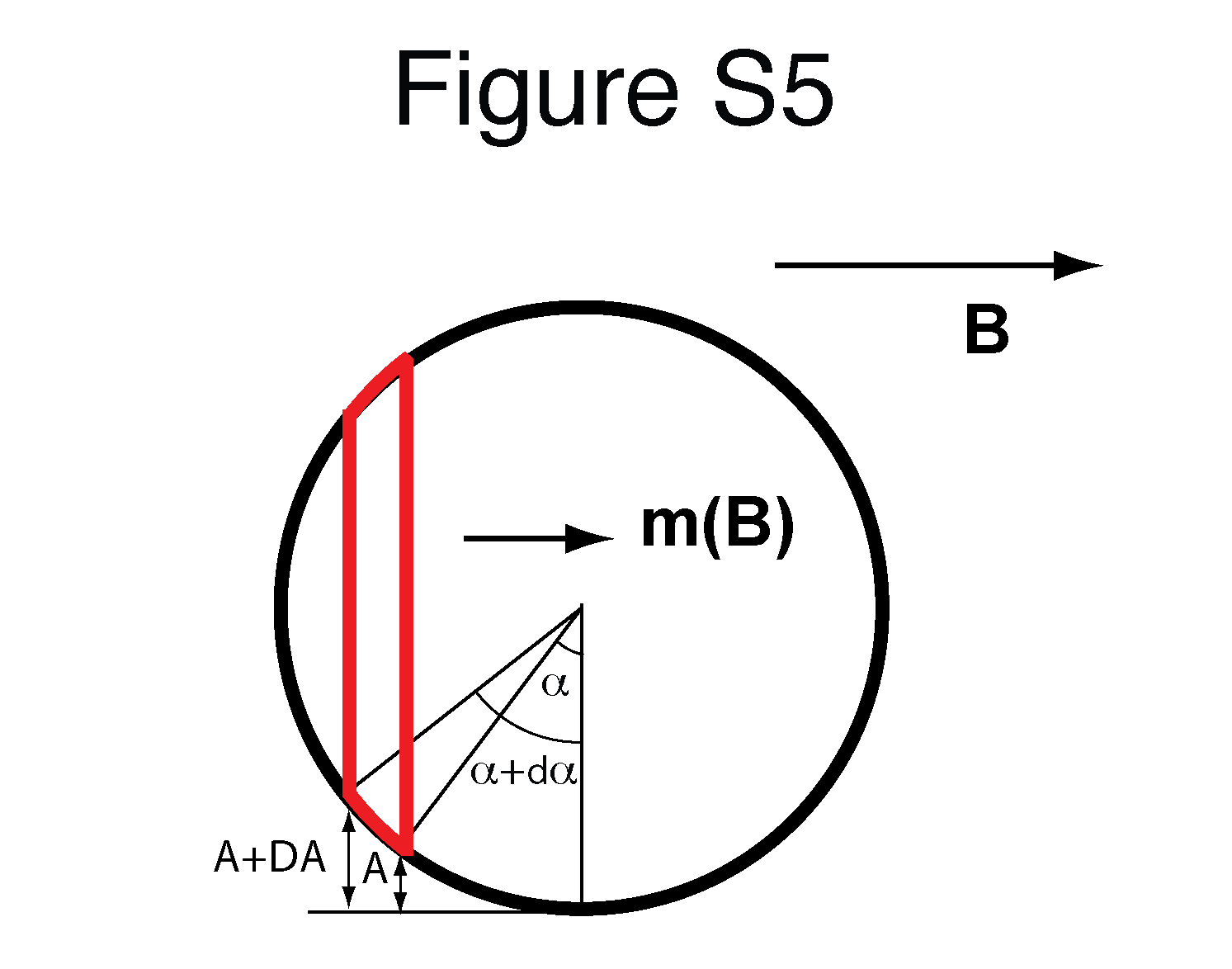


**Figure S5.** Schematic of bead indicating geometric parameters used to calculate the probability density function of DNA attachment offsets. The bead has a preferred axis in paramagnetic polarizability that aligns along the direction of the magnetic field. The bead is free to rotate about the magnetic field axis. Molecules that bind to the segment of the sphere marked in red and defined by angles α and α + dα lead to a bead attachment offset in the range A to A + ΔA.

## 3D modeling of force fields in magnetic tweezers

### Formalism and parameters

Here, we discuss the formalism used to calculate the magnetic fields generated by the magnet pair used in the experiments. We use gold-plated (Ni-Cu-Ni-Au), 5 x 5 x 5 mm neodymium-iron-boron (NdFeB) permanent magnets (W-05-N50-G, supermagnete, Uster, Switzerland). The manufacturer quotes a residual magnetism, also known as the macroscopic magnetic moment density **M**, of 1.4-1.46 T (<http://www.supermagnete.de/eng/data_table.php>). However, previous measurements by Lipfert et. al. suggest that the value is closer to 1.24 T, which is the value used in this study. We calculate the magnetic fields generated by a pair of such magnets, arranged in a vertical configuration (see Fig. S6 and ) and separated by a gap, g = 2 mm. The magnetostatic problem at hand is modeled by introducing a uniform magnetic surface charge density, σM, on the bottom and top planes of the magnets (see Fig. S6a). The basic equations of magnetostatics read:

$\nabla\cdot\mathbf{B}=0$, (9)

$\nabla\times\mathbf{H}=\mathbf{J}$, (10)

with **J** a current density, and **B** and **H** are magnetic fields related by **B** = μ0(**H**+**M**), where **M** is the macroscopic magnetic moment density of the material. For a finite region in space with vanishing current density,$\nabla\times\mathbf{H}=0$, and a magnetic scalar potential, ФM, can be introducedsuch that $\mathbf{H}=-\nabla\varphi_{M}$**.** Given Eq. 9, $\nabla\cdot\mathbf{H}=-\nabla\cdot\mathbf{M}$, a magnetostatic analog of the Poisson equation can be introduced:

$\nabla^{2}\varphi_{M}=-\rho_{M}$. (11)

Here, the effective magnetic charge volume density is given by$\rho_{M}=-\nabla\cdot\mathbf{M}$. The magnetic potential is then calculated following:

$\varphi_{M}(\mathbf{r})=\frac{1}{4\pi}\int\frac{\rho_{M}(\mathbf{r}')}{|\mathbf{r}-\mathbf{r}'|}d^{3}r'$ (12)

For a uniform magnetization throughout the volume of the magnets, it can be shown that eq. 12 reduces to :

$\varphi_{M}(\mathbf{r})=\frac{1}{4\pi}\oint_{S} \frac{\sigma_{M}}{|\mathbf{r}-\mathbf{r}'|}dA$. (13)

σM is given by **M∙n** where **n** is the unit vector normal to the surface of the magnet. Thus the top and bottom surfaces of the magnet have surface charge density σM of equal magnitude (1.24/µo A/m) and opposite sign.

The magnetic moment induced in the superparamagnetic bead as a function of the magnetic field, $\mathbf{m}(\mathbf{B})$, is calculated using:

$M(B)=M_{sat}(\coth(B/B_{0})-\frac{1}{B/B_{0}})$. (14)

where Msat and B0 are the saturation magnetization and the characteristic field of the superparamagnetic beads respectively (Msat = 43.3 kA/m and B0 = 12 mT quoted by the vendor). We used an empirically determined scaling factor (factor 1.4 as in ) to account for discrepancies between predicted and measured magnetic forces exerted on MyOne paramagnetic beads, that is most likely due to an underestimation of these vendor-quoted magnetization values.

The force, **F** experienced by the superparamagnetic field can then be calculated as:

$\mathbf{F}=\frac{1}{2}\nabla(\mathbf{m}(\mathbf{B})\cdot\mathbf{B})$. (15)

Using the above formalism, we have numerically calculated the magnetic field distribution and the force field distribution as function of distance of the bottom of the magnet pair to the top of the flow cell, Zmag. Equations were solved and numerically evaluated with Mathematica 7.0. All calculations referred to in the paper and in the supplemental section were performed for a magnet pair with a magnet-to-magnet distance of 2 mm and a vertical magnet configuration (see sketch in Fig. S6a). We furthermore take into account a vertical spacing between the top of the flow cell and the magnetic beads tethered to the top of the bottom glass slide of the flow cell of 0.4 mm.

###

## Supporting References

1. Leach J, Mushfique H, Keen S, Di Leonardo R, Ruocco G, et al. (2009) Comparison of Faxen's correction for a microsphere translating or rotating near a surface. Physical Review E 79: 026301.

2. Bouchiat C, Wang MD, Allemand JF, Strick T, Block SM, et al. (1999) Estimating the Persistence Length of a Worm-Like Chain Molecule from Force-Extension Measurements. Biophysical journal 76: 409-413.

3. Klaue D, Seidel R (2009) Torsional Stiffness of Single Superparamagnetic Microspheres in an External Magnetic Field. Physical Review Letters 102: 028302.

4. Lipfert J, Hao X, Dekker NH (2009) Quantitative Modeling and Optimization of Magnetic Tweezers. Biophysical journal 96: 5040-5049.

5. Jackson JD, Fox RF (1999) Classical electrodynamics. American Journal of Physics 67: 841.
